# Assessment of metabolic phenotypic variability in children’s urine using ^1^H NMR spectroscopy

**DOI:** 10.1038/srep46082

**Published:** 2017-04-19

**Authors:** Léa Maitre, Chung-Ho E. Lau, Esther Vizcaino, Oliver Robinson, Maribel Casas, Alexandros P. Siskos, Elizabeth J. Want, Toby Athersuch, Remy Slama, Martine Vrijheid, Hector C. Keun, Muireann Coen

**Affiliations:** 1 ISGlobal, Centre for Research in Environmental Epidemiology (CREAL) Barcelona, Spain; 2 Universitat Pompeu Fabra (UPF), Barcelona, Spain; 3 CIBER Epidemiología y Salud Pública (CIBERESP), Madrid, Spain; 4 Division of Computational and Systems Medicine, Department of Surgery and Cancer, Faculty of Medicine, Imperial College London, London, SW7 2AZ, UK; 5 Division of Cancer, Department of Surgery and Cancer, Imperial College London, Institute of Reproductive and Developmental Biology (IRDB), Hammersmith Hospital, London W12 0NN, UK; 6 MRC-PHE Centre for Environment and Health, School of Public Health, Faculty of Medicine, Imperial College London, London, W2 1PG, UK; 7 Inserm, Univ. Grenoble Alpes, CNRS, IAB (Institute of Advanced Biosciences), Team of Environmental Epidemiology applied to Reproduction and Respiratory Health, F-38000 Grenoble, France

## Abstract

The application of metabolic phenotyping in clinical and epidemiological studies is limited by a poor understanding of inter-individual, intra-individual and temporal variability in metabolic phenotypes. Using ^1^H NMR spectroscopy we characterised short-term variability in urinary metabolites measured from 20 children aged 8–9 years old. Daily spot morning, night-time and pooled (50:50 morning and night-time) urine samples across six days (18 samples per child) were analysed, and 44 metabolites quantified. Intraclass correlation coefficients (ICC) and mixed effect models were applied to assess the reproducibility and biological variance of metabolic phenotypes. Excellent analytical reproducibility and precision was demonstrated for the ^1^H NMR spectroscopic platform (median CV 7.2%). Pooled samples captured the best inter-individual variability with an ICC of 0.40 (median). Trimethylamine, *N*-acetyl neuraminic acid, 3-hydroxyisobutyrate, 3-hydroxybutyrate/3-aminoisobutyrate, tyrosine, valine and 3-hydroxyisovalerate exhibited the highest stability with over 50% of variance specific to the child. The pooled sample was shown to capture the most inter-individual variance in the metabolic phenotype, which is of importance for molecular epidemiology study design. A substantial proportion of the variation in the urinary metabolome of children is specific to the individual, underlining the potential of such data to inform clinical and exposome studies conducted early in life.

Metabonomic analysis explores the integrated response of an organism to environmental changes. Increasing evidence points toward the critical and long-term involvement of early life environmental exposures and lifestyle on later health and disease risk predisposition^1^. Metabolic profiling is now a well-established top–down systems biology approach for characterising the role of metabolism in gene–environment–health interactions^2^. The generalisation of such approaches and systematic prospective collection of blood and urine samples in large mother-child cohorts opens up new research opportunities for understanding and discovering the impact of pre-natal and post-natal exposures on the onset of child and adult physiological conditions. Numerous metabolic phenotyping studies have investigated the impact of anthropometric factors such as age, sex, and obesity in an attempt to understand the human metabolome and inter-individual variance^3^,^4^. These studies have mainly been conducted on adults, whereas studies on children or adolescents are rare. This population requires specific assessment of variance in metabolic phenotypes due to rapid developmental changes, differential lifestyle patterns and age-dependent response to environmental factors. Indeed, exposures during the pubertal physiological window may be responsible for the later appearance of several metabolic conditions^5^. In addition, data on short-term temporal variability of metabolic phenotypes of repeat urine samples are lacking. One of the major concerns over the predictive potential of metabolic phenotyping in the clinic is the temporal variability of the metabolome^6^,^7^. A study with access to repeat sampling under controlled conditions with human subjects showed that 22% of the identified metabolites in ^1^H NMR urinary spectroscopic profiles exhibited a significant 24 h cosine rhythm^7^. This effect is believed to be particularly marked in the morning in comparison to the rest of the day. This reflects a more pronounced influence on homeostasis when food is consumed after an 8 h fast as compared to a shorter inter-meal time interval during the day, which may be due to circadian influences independent of meal consumption^6^. It has also been shown in in-patient studies with tightly controlled environmental conditions, that diet or day-to-day variability do not account for the largest source of variability in either blood or urine metabolic profiles^8^,^9^.

Even without specific consideration of diurnal effects, studies which have investigated the utility of metabonomic approaches in large adult populations showed that 60% (plasma) and 47% (urine) of biological variation in ^1^H NMR-detectable metabolite concentrations was stable and representative of familial and individual-environmental factors^10^. Other recent studies suggest that the stable component of inter-individual variation or intraclass correlation (ICC), over a four-month to a year interval was between 0.43 to 0.57^10^,^11^. Thus the current literature supports the notion that metabolic phenotypes in biofluids are stable over the short to medium term in adults, however, equivalent evidence is lacking for children’s biofluids. The unstable component of a given metabolic phenotype will complement the study of the stable component, and provides a window into the systemic response to acute environmental perturbations, such as dietary change or exposures.

In this study we focus on ^1^H NMR spectroscopic analyses of urine, which in comparison to blood is a non-invasive biofluid to access, making it a more attractive choice for large-scale biological sampling in children. We examined the influence of the sample collection time-point of the day on the metabolic phenotype, assessed analyte detectability and quantification, and the likely sources of short term variability within and between children. We confirm the high analytical reproducibility and robustness of NMR-based urinary metabolic phenotyping (as reported elsewhere, ref. 12) and illustrate the benefits of pooling spot urines when seeking the stable component of the metabolome.

## Results

**Figure 1:**
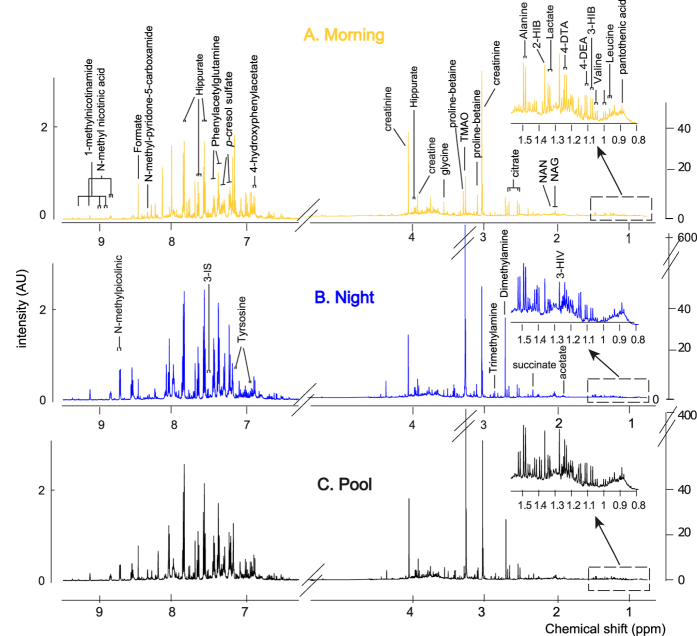
A typical ^1^H NMR spectrum of urine from an 8 year old male child collected in the morning (**A**), night-time (**B**) and pooled (C; 50:50 morning and night-time) with identified metabolites. Abbreviations: 2-HB, 2-hydroxybutyrate; 3-HB, 3-hydroxybutyrate; 3-HIV, 3-hydroxyvalerate, 3IS, 3-indoxylsulfate; 4-DEA, 4-deoxyerythronic acid; 4-DTA, 4-deoxythreonic acid; NAG, *N*-acetyl glycoprotein fragments; NAN, *N*-acetylneuraminic acid; TMAO, trimethylamine-N-oxide.

First morning void and night-time urinary samples were collected from 20 healthy Caucasian children (8–9 years, 6 females and 14 males), over a period of six days, and pooled 50:50, generating 324 samples in total (36 samples were missing randomly across the children and the days, leaving 108 triads with complete morning, night and pooled samples for each day). Representative ^1^H NMR spectra of urine samples obtained from an 8-year old in the morning (A), night-time (B), and pooled (C; 50:50 morning and night-time samples) are shown in Fig. 1, indicating the coverage of the urinary metabolome in this study.

### Analytical variability

As a first step in the characterisation of the stability of child metabolic phenotypes, analytical variability of NMR data was assessed to inform on the robustness and stability of the NMR platform. Repeat analysis of a representative pooled sample (quality control, QC sample) was conducted. The percentage coefficient of variation (CV%) of the QC sample was calculated for integrals of NMR signals representing individual metabolites (raw integral divided by the internal reference, before creatinine normalisation) and showed an average CV% of 7.2% and median 7.7%. Metabolites with a low signal to noise ratio (S/N) in QCs, such as *N*-methylpicolinic acid, 3-aminoisobutyrate, *N*^1^-methylnicotinamide and acetone, presented a CV% over 10%. Other metabolites, such as lysine, hippurate, trimethylamine-N-oxide (TMAO), showed the best analytical stability with a CV% below 3%. The CV% for all identified metabolites (n = 44), are presented in Table 1 along with the representative resonance integration windows selected for each metabolite and signal to noise (S/N) ratio.

As a complementary approach to assess analytical and sample processing variability in the biological samples, the difference between the daily pool and the average of the morning and the night sample were calculated (named %DiffPool, based on 108 paired morning and night samples, raw integral divided by the internal reference). The %DiffPool showed an average of 8.7% and a median of 5.4% with more variability captured than the CV calculated with the standardised QC samples for certain metabolites such as *N*-methylpicolinic acid, 3-aminoisobutyrate, proline betaine, succinate, acetone and carnitine. A detailed table of the metabolite differences across morning and night samples per day and per individual also suggests that these six metabolites showed the most variability across morning/night samples but with a high intra-day and inter-individual variability based on visual inspection of the ratios (SI Table 4).

### Quantification of urinary metabolites

We quantified urinary metabolites based on peak integrals and to account for the short recycle time of the NMR acquisition, and hence incomplete relaxation of ^1^H nuclei, the longitudinal relaxation time, T_1_, was calculated for each metabolite and a correction factor was applied to resulting integrals (SI Table 1). Of the 44 identified metabolites, 18 of them were of low abundance or were only detected in a subset of samples, and therefore reliable estimation of their longitudinal relaxation time (T_1_) was not possible (semi-quantification). The final concentration estimates for metabolites (n = 26) are presented in Table 2. Creatinine was the most abundant detectable metabolite, with a mean concentration of 5.95 (IQR 4.73–7.24) mmol/L, whereas isoleucine was the least abundant metabolite that could be reliably quantified with a mean concentration of 2.0 (IQR 1.6–2.3) μmol/mmol of creatinine in the daily pooled samples. Most metabolites displayed a large dynamic range, particularly TMAO, hippurate and creatine with order of magnitude differences between minimum and maximum concentration values.

### Biological short-term variability in the ^1^H NMR spectroscopic metabolic profiles of morning, night-time and pooled urine samples

**Figure 2:**
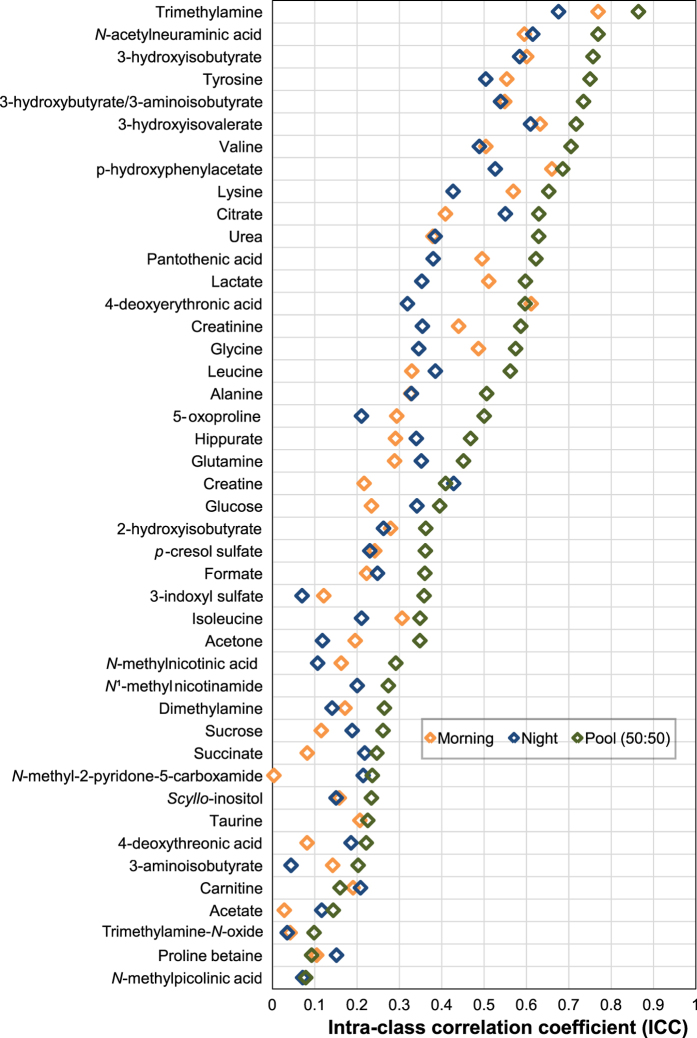
Short term variability over six days for 44 metabolites in morning, night-time and pooled urine samples based on intra-class correlation coefficients (ICCs) measured in 20 children by ^1^H NMR spectroscopy. Each child was sampled twice daily over a period of one week (morning and night-time).

A key objective of the study was to ascertain whether the inter-individual variability captured in the urinary NMR-based metabolic phenotype was greater than the intra-individual variability assessed across six days. We simultaneously sought to define which type of urine sample (morning, night or pool) captured best this inter-individual variability, as this is of relevance in molecular epidemiology studies for both study design and biological interpretation. The variability of urinary metabolites across six days was assessed independently in morning, night-time and pooled samples based on the intra-class correlation coefficient (ICC) (Fig. 2). Metabolites were normalised by creatinine for comparison purposes with other studies. We also applied probabilistic quotient normalisation^13^ to our data, and this showed similar biological variability results to the normalisation by creatinine (results not shown). Pooled samples captured the best inter-individual variability with 19 out of 44 metabolites with ICC values above 0.5 whereas only 11 and 8 metabolites were above ICC 0.5 respectively in morning and night-time samples.

Trimethylamine, *N*-acetyl neuraminic acid, 3-hydroxyisobutyrate, 3-hydroxybutyrate/3-aminoisobutyrate, tyrosine, valine, 3-hydroxyisovalerate were the metabolites that showed the least intra-individual variability with ICCs in pooled samples over 0.7, whereas TMAO, proline betaine, acetate, *N*-methylpicolinic acid were the least stable with ICCs under 0.2 (Fig. 2).

**Figure 3:**
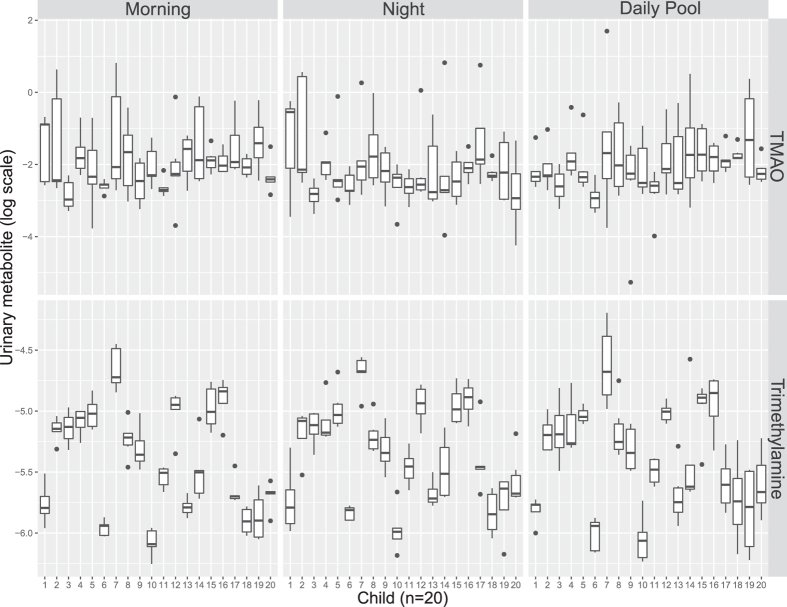
Distribution of urinary trimethylamine-N-oxide TMAO (top panel) and trimethylamine (bottom panel) based on ^1^H NMR spectra of urine samples obtained from 20 children across morning, night-time and pooled samples (50:50 morning and night-time samples). Metabolite integrals were log transformed. TMAO, Trimethylamine-N-oxide. A.U. arbitrary units.

Figure 3 clearly shows that TMAO concentration greatly varies over 6 days with overlapping distributions across the twenty children. In contrast the precursor of TMAO, trimethylamine, was the most stable metabolite measured, with a characteristic concentration range for each child, tightly controlled over six days with limited overlap across the children.

**Figure 4:**
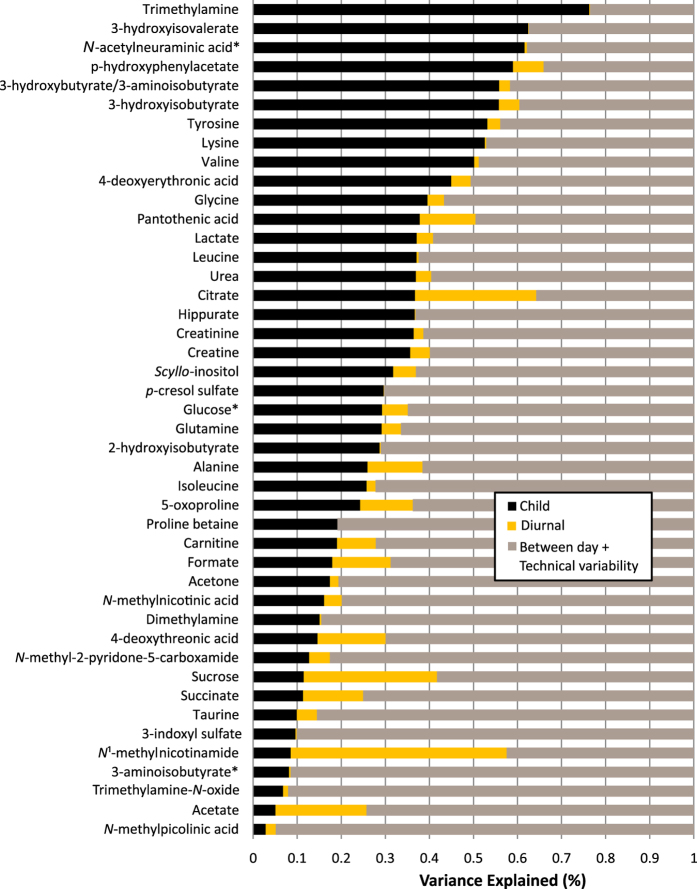
Decomposition of variance for each annotated metabolite resonance in ^1^H NMR spectra. The plot displays estimates for the proportion of biological variance explained by child characteristic (black) and diurnal (yellow) components. The remainder of the variance is attributed to day-to-day and technical variability and unknown sources (residual, brown). Metabolites are ordered by estimated child specific variance. *Multiple overlapping resonances. Integral assigned to the most likely, abundant metabolite.

The total variance of each metabolite was decomposed according to the longitudinally stable or child specific variation (black), diurnal variation (yellow) and residual variation (brown, comprised of technical variance, between-day variance and unknown) (Fig. 4). The proportion of stable variation representative of the inter-individual variability showed a large range depending on the metabolite, with an average value of 24%. Trimethylamine, short chain fatty acids including 3-hydroxyisovalerate, 3-hydroxyisobutyrate, 3-hydroxybutyrate/3-aminoisobutyrate, *p*-hydroxyphenylacetate and some amino acids i.e. tyrosine, lysine and valine, exhibited the highest stability with over 50% of variance donor specific. Among the metabolites with a residual variance over 80%, are *N*-methylpicolinic acid, TMAO, dimethylamine, taurine and *N*-methylnicotinic acid which also displayed a high dynamic range in urinary levels. Other metabolites with a high residual variance such as succinate, 3-aminoisobutyrate, acetone and acetate also had a high analytical variability in QC samples (CVqc). A few metabolites presented a large diurnal variation, in particular *N*-methylnicotinamide, sucrose, citrate and acetate (20–47% of total variation explained by morning/night sampling).

Further description of the diurnal variation in urinary metabolites show that 15 out 44 metabolites were significantly different between morning and night, after accounting for multiple testing (Bonferroni correction; p < 0.001). *N*-methylnicotinamide was particularly increased in morning samples (+53% [IQR 25;71%]) compared to the night-time samples (SI Table 2). Sucrose and citrate were lower in morning samples; −148% [IQR −580;−15%] and −52% [IQR −110; −14%] respectively, with a large interquartile range across individuals. Diurnal changes at the individual and day level are presented in SI Table 4.

Gender differences were also characterised using univariate statistics. After accounting for multiple testing (Bonferroni correction; p < 0.001), the concentration of 28 of the 44 measured metabolites were different between females (n = 6) and males (n = 14). Specifically, metabolic phenotypes of males showed the presence of significantly higher concentrations of tyrosine, formate and lysine (respectively +38%, +40%, +43%) and lower creatinine and deoxythreonic acid compared to females (see SI Table 3 for full non-parametric Mann–Whitney U-test results).

## Discussion

Our study characterises for the first time short-term temporal variability of the urinary metabolome in children. A detailed understanding of such short-term temporal variability and behaviours that influence metabolic phenotypes at the individual level enhances their utility in a variety of clinical, epidemiological and occupational contexts. We characterised the longitudinal variation in urinary metabolite profiles obtained from children (n = 20) twice a day for six days, using ^1^H NMR spectroscopy. It was possible to decompose the metabolic phenotypic diversity observed, and determine stable and unstable temporal variation over six days. In addition, we provided evidence of diurnal variation of metabolic signatures and proposed analysis of a pooled urine sample in order to capture the largest inter-individual variability. These results can inform on measurement uncertainties for use in larger cohort analysis and correct exposure/outcome models based on this error.

Excellent analytical reproducibility and precision (median CV%s 7.2% across all metabolites in repeat analysis of a quality control pooled sample) in our study provides a strong position for subsequent assessment of intra- and inter-individual variability of metabolic phenotypes. ^1^H NMR profiling provided a broad metabolic coverage with phenotypes exhibiting high stability for 18 metabolites (ICC > 0.5) that included trimethylamine, *N*-acetyl neuraminic acid, 3-hydroxyisobutyrate, 3-hydroxybutyrate/3-aminoisobutyrate, tyrosine, valine, 3-hydroxyisovalerate. Ideally for a metabolite to be validated as a clinical or exposure biomarker, the analytical variability and the intra-individual variability must be smaller than the inter-individual variability. The effect size for diagnostic purposes should be nested in the inter-individual variability and the residual variability due to between day and analytical variability must be smaller than the smallest significant change in metabolite levels associated with the phenotype or outcome of interest.

Using mixed effect model analysis-of-variance techniques we quantified the stable proportion of between-person variability across six days together with diurnal variation. Our results strongly corroborate earlier findings^14^,^15^,^16^,^17^,^18^ including Nicholson *et al*. which simultaneously estimated familial, individual-environmental, short-term dynamic (visit), and non-biological variation in an adult twin study design^10^. In that study, trimethylamine showed the strongest familial component whereas hippurate exhibited the strongest stable environmental component; both observations confirming a high stability over time. Low inter-individual variability (under 10%) for *N*-methylnicotinic acid and TMAO in our study can easily be explained by dietary influences. Urinary TMAO levels are highly related to consumption of foods that contain TMAO (fish) or its dietary precursors, choline, betaine and carnitine (eggs and beef) and to gut microbial activity^19^,^20^. The high variability observed for urinary *N*-methylnicotinic acid may also result from differing patterns of food intake in children, particularly related to coffee or potentially soda drinks and chocolate intake^21^,^22^. Other metabolites we identified as highly stable within individuals such as *p*-hydroxyphenylacetate, 3-hydroxyisovalerate, 3-hydroxybutyrate/3-aminoisobutyrate were not reported in previous longitudinal variation studies because of limited assignment and possibly because of population demographic differences - for example the Nicholson *et al*. study only investigated post-menopausal females. Stable markers such as 3-hydroxyisovalerate and *N*-acetyl neuraminic acid (also called sialic acid, putatively annotated in our data based on the *N*-acetyl signal) should be considered in clinical settings since there is evidence they may serve as markers of immune-mediated inflammatory diseases (IMIDs), a group of complex and prevalent diseases where prognostic monitoring is highly challenging^23^,^24^. However, metabolites with high variance across six days such as taurine, *N*-methylpicolinic acid, or during the day such as sucrose, possibly following dietary intake, could be of interest for nutritional epidemiology studies.

Results on sex differences such as increased creatinine excretion in females are different to previous findings in adults where creatinine usually correlates with muscle mass^25^. However, this result, as well as higher citrate excretion in females, corroborates a previous study on sex differences in children of 12–15 years old^26^ and adults^27^. These results should be corroborated in a larger study.

Future epidemiological studies may choose to analyse morning/night pooled samples in order to capture the best inter-individual variability over intra-individual variability. ICCs for pooled samples in our study suggest a higher reliability of the urinary metabolite excretion data compared to previous NMR-based metabonomic studies where ICCs of 30–37% were found on average for metabolites present across two 24-hour urine collections^3^. However, the INTERMAP population was substantially larger (n > 2300) and more geographically diverse (inter-continental). Our findings are closer to those reported by Floegel and colleagues who found that the median ICC over a 4-month interval for 163 sera metabolites measured by mass spectroscopy was 0.57^27^.

Diurnal variation in urinary excretion affects only a subset of metabolites which are related to known physiological processes and dietary intake. Increased *N*-methylnicotinamide and creatinine in morning samples compared to night and decreased concentrations of citrate, sucrose, taurine, creatine are in agreement with previous studies^7^,^16^,^18^. Interestingly, sucrose which increases in night-time samples in our study, likely due to dietary intake, was proposed as a marker of sugar intake in an obese population^28^. Other metabolites are established to vary throughout the day due to physiological processes such as creatinine which fluctuates depending on glomerular filtration rate and physical activity^29^,^30^. *N-*methyl nicotinamide in several studies was also shown to be high in the morning before breakfast, probably due to reduced enzymatic activity following fasting^31^.

Overall, the effects of inter-/intra-individual differences on the child urinary metabolome observed in this study are very similar to the ones previously observed in adults. Few studies have assessed metabolic variability in children/adolescents^26^,^32^,^33^. Strong age effects in the first years of life have been identified in a PCA scores plot based on urine ^1^H NMR spectroscopic profiles from 55 children from newborns to 12 years old^31^ and related to growth spurt during early childhood^33^. Sex and pubertal development (Tanner stage) were characterised clearly in 12–15 year old children based on metabolic profiles^26^. Metabonomics in paediatric populations has mainly found applications in respiratory diseases, neuro-developmental and obesity outcomes^34^,^35^,^36^. However, studies assessing the long term stability of the metabolic milieu which represents dietary intake, lifestyle and genetic factors, and disease risk factors would be of interest. Using the metabolite profile of healthy children as a phenotyping tool has great potential to measure the impact of early environmental exposures. Indeed children are more susceptible to their environment including factors such as infections, gut microbial variation, pollutants and may undergo physiological disturbances which do not display clinical symptoms until adulthood. The HELIX project will use information on the child metabolome across six different European countries to characterise the burden and effect of environmental exposures^37^.

### Limitations

While the current study design did not directly address long-term stability beyond six days, the rate and nature of the changes in metabolic stability is an interesting topic for further research and will be facilitated as biobanks grow, providing samples for cohort studies capable of characterizing very long-term molecular variation.

The modest sample size of the sub-cohort characterised in our study (n = 20), did not allow for an analysis of further phenotypic variations such as adiposity or relation to environmental exposures. Future work in the HELIX study will address this need by allowing comprehensive characterisation of children’s metabolic variability in combination with in depth environmental exposure assessment and additional ‘omics analyses (proteomics, transcriptomics and epigenomics). Further information on daily dietary intake, physical exercise or physical stressors are needed to investigate the relative contribution of lifestyle and circadian rhythm to variance in the metabolic phenotype.

## Methods

### Study population

The Human Early-Life Exposome (HELIX) project aims to integrate novel exposure assessment and ‘omics’ technologies to characterise early-life exposure to multiple environmental factors and associate these with child health outcomes^37^. HELIX comprises 6 existing birth cohort studies (32,000 mother-child pairs) across Europe, of which 1,200 mother-child pairs were selected for phenotyping including exposure and ‘omics signatures. Smaller nested panel studies (n = 150 children and pregnant mothers) collected in-depth personal exposure data and repeat biological samples for a weekly period across two seasons. This study focused on a subset of 20 healthy children from the Spanish part of the panel study, nested with the INMA (INfancia y Medio Ambiente) cohort in Spain^38^.

Research has been carried out according to the international and national guidelines and regulations (including the declaration of Helsinki). Specifically for Spain, the Spanish Law on Biomedical Research (14/2007, of 3rd July). All research protocols were approved by the PS-Mar Ethics Committee (N° 2005/2106/I). Informed consent was obtained from all subjects.

### Urine sample collection and preparation

Two urine samples per day, first morning void and night-time, were collected for six days in 70 ml polypropylene containers. Families recorded the date and time of each collection prior to storage in a domestic freezer (typically −20 °C). On day 7, samples were transported from each family residence to the analytical laboratory in a −80 °C freezer, with thawing in transit prevented using cool box ice packs. Urines of each child were aliquoted together: urines were defrosted overnight at 4 °C and placed at room temperature 30 min before aliquoting. Urines were inverted gently 2–3 times and from each sample three aliquots of 1.75 ml in a 2 ml cryovial were made. Aliquots of individual paired daily morning (n = 108) and night-time (n = 108) urine collections were pooled (n = 108) to permit a comparison with single morning/night-time urinary collections. The total biological sample size was 344 urines, which included morning, night and daily pooled samples. Fifteen samples could not be analysed as a consequence of insufficient sample volume or missed collection. Aliquots were then stored at −80 °C until shipment to Imperial College London.

Prior to analysis, samples were thawed and homogenised using a vortex mixer. They were centrifuged at 13,000 *g* for 10 min at 4 C to remove insoluble material. 600 μL of each urine sample was transferred into 96-well plates for NMR spectroscopy using a Bruker Sample Track system and a Gilson Liquid Handler 215 preparation robot. The robot mixed 540 μL of sample with 60 μL of a buffer solution (1.5 M KH_2_PO_4_, 2 mM NaN_3_, 1% 3-(trimethylsilyl)-[2,2,3,3-*d*_4_]-propionic acid sodium salt (TSP-d_4_) solution, pH 7.4) and placed it in an NMR tube (5 mm Bruker SampleJet NMR tubes).

Quality control (QC) samples were prepared to monitor analytical variability of the metabolic profiling platform. A pooled urine QC sample was prepared by mixing 200 μl of each individual sample of the study (~60 ml total volume). The pooled QC sample was aliquoted into cryovials and stored at −40 °C for all future analyses. A total of 24 QC samples were included in the analytical run, spaced at regular intervals (every 30 samples, four newly prepared QC per analytical batch/well plate). To assess stability of one QC sample over time, this was analysed twice at the beginning and the end of every well plate (4 repeats in total per well plate).

### ^1^H NMR spectroscopy analysis

One-dimensional 600 MHz ^1^H NMR spectra were acquired on a BrukerAvance III spectrometer operating at 14.1 Tesla, equipped with a 5 mm broad-band inverse configuration probe maintained at 300 K and BrukerSampleJet system with well plates kept at 6 °C. The ^1^H NMR spectra were acquired using a standard one-dimensional solvent suppression pulse sequence (relaxation delay, 90° pulse, 4 μs delay, 90° pulse, mixing time, 90° pulse, acquire FID). For each sample, 128 transients were collected into 64 K data points using a spectral width of 12,000 Hz with a recycle delay of 4 s, a mixing time of 100 ms, and an acquisition time of 2.73 s. A line-broadening function of 0.3 Hz was applied to all spectra prior to Fourier transformation. All ^1^H NMR spectra were automatically phased and baseline-corrected using Topspin 3.2 software (BrukerBioSpin, Rheinstetten, Germany). The ^1^H NMR spectra of urine were referenced to the TSP-*d*_4_ resonance at 0 ppm.

### Data processing

NMR spectra were imported into the Matlab 2014a (MathWorks, Massachusetts, US) computing environment, and were aligned using the recursive segment-wise peak alignment method, an algorithm based on cross-correlation^39^. The pool QC sample spectrum was used as reference for alignment. A single representative resonance in the spectrum was selected for each assigned metabolite, based on presence in a high proportion of the spectra, with high signal-to-noise ratio, and exhibiting limited overlap with other resonances. Metabolite resonance peak areas were estimated using trapezoidal numerical integration and 44 metabolites were obtained using this method. Signal-to-noise ratio to noise ratio (S/N) was calculated with the raw integral for a given peak by calculating the root mean square (RMS) noise using a representative noise region of the spectrum (9.5–9.9 ppm).

### Metabolite quantification using in-house integration routine for a subset of metabolites

The concentration of a given metabolite can then be estimated from the signal of the internal standard of known concentration, TSP-*d*_4_, using the following formula:


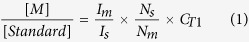


where [*M*] is the metabolite molar concentration, [*Standard*] is the known molar concentration of internal standard TSP, *I*_*m*_ is the metabolite integral, *I*_*S*_ is the integral of the TSP-d4 peak, *N*_*m*_ is the number of ^1^H nuclei contributing to the metabolite peak, and *N*_*s*_ is the number of ^1^H nuclei contributing to the internal standard’s peak. *C*_*T1*_ is the compensation factor for incomplete longitudinal relaxation. And


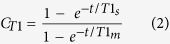


where t, total time per transient, is the sum of recycling delay time and acquisition time of the pulse sequence, *T1*_*m*_ and *T1*_*s*_ are respectively the T_1_ (longitudinal relaxation time) of the metabolite and the TSP-*d*_4_ resonance as measured using a standard inversion recovery experiment. For the standard inversion-recovery pulse sequence experiment (180° pulse – τ – 90° pulse), variable relaxation delay, τ, was chosen logarithmically to cover values from 0.001 to 5 seconds. For each τ, 4 transients were collected into 64 K data points using a spectral width of 12,000 Hz with a recycle delay of 32 seconds^40^. T_1_ values are presented in SI Table 1.

Of the 44 identified metabolites, 18 of them were of low abundance or were only detected in a subset of samples, and therefore reliable estimation of their longitudinal relaxation time (T_1_) was not possible.

Metabolites were included in the subsequent data analyses based on quantified values (n = 26) and semi-quantified integral values (n = 18) and were normalised to creatinine. Finally, metabolite levels were log–transformed for the ICC calculation and the decomposition of variance analysis, in order to meet assumptions of normality and homoscedasticity in statistical tests and to minimise the influence of extreme values.

### Metabolite annotation

Assignment of endogenous urinary metabolites was made by reference to published literature data^41^,^42^, online databases (HMDB)^43^, statistical total correlation spectroscopy (STOCSY)^44^ and using ChenomxNMRsuite profiler (ChenomxInc, Edmonton, Canada) and/or confirmed by 2D NMR experiments on a selected sample including homonuclear ^1^H-^1^H correlation spectroscopy (COSY), ^1^H-^1^H total correlation spectroscopy (TOCSY) and ^1^H-^13^C NMR heteronuclear spectroscopy (sample selected based on high abundance of doublet at 8.72 ppm). Spike-in experiments using authenticated chemical standards were required for certain final metabolite annotations.

### Statistical analyses

To determine the reproducibility of the NMR platform we computed the CV (coefficient of variation, the standard deviation divided by the mean) for a subset of metabolites identified in the QC samples which were interspersed across the NMR run. An additional measure of reproducibility was calculated for each metabolite based on the % difference between the average of the Morning and Night samples, and the Daily Pool (50:50) sample in Sample ***i**, Z*_*i*_:


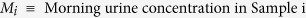



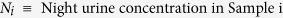



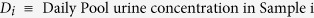



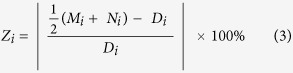


%DiffPool is the median value of {*Z*_*i*_} with n being the number of data pairs (6 days × 20 individuals minus missing pairs, total n of 108 samples). This represents the typical measurement error in the difference of the pooled sample from the average of the morning and night samples.

Intraclass correlation coefficients (ICC) were calculated to investigate the repeatability of metabolites across the six day study period independently in pooled, morning and night-time samples. ICC is a measure of the reliability of repeated measures over time, defined as the ratio of between-subject variance to total (between-subject plus within-subject). Between and within-subject variance was calculated from the mean squares in the analysis of variance using one-way ANOVA fixed effects model using ‘psych’ R package ‘ICC’ function (from CRAN repository). We report the ICC for Single_raters_absolute^45^.

The relative importance attributed to between-individual and diurnal variations were estimated across all metabolites based on variance decomposition models. Using linear mixed effect models, by-subject random slopes were modelled for within-subject factors. The time of the day (morning/night; diurnal) was added as a fixed effect. This regression model was calculated for each metabolite using the R package ‘lme4’ and the function lmer^46^. The proportion of variance explained, or multiple R^2^, was calculated as ratios of the total variance and represented as the per cent of variability because of differences between children. In addition, the residual variance contains the between day and technical variability but these factors were not added to the model.

## Additional Information

**How to cite this article**: Maitre, L. *et al.* Assessment of metabolic phenotypic variability in children’s urine using ^1^H NMR spectroscopy. *Sci. Rep.* **7**, 46082; doi: 10.1038/srep46082 (2017).

**Publisher's note:** Springer Nature remains neutral with regard to jurisdictional claims in published maps and institutional affiliations.

## Supplementary Material

Supplementary Information

## Figures and Tables

**Table 1:** Analytical variability in urinary metabolites measured in ^1^H NMR spectra in 24 repeat urine samples (pooled representative QC sample) and in 50:50 pool samples.

Metabolite	resonance signal (ppm)	integrated region		Signal/Noise ratio (log10)	CV (Quality control sample, N = 24)	%DiffPool (50:50 samples, N = 108)	ChEBI ID**
		lower bound (ppm)	upper bound (ppm)				
2-hydroxyisobutyrate	1.36 (s)	1.362	1.366	3.57	7.3%	3.9%	19641
3-aminoisobutyrate*	2.63 (m)	2.617	2.633	2.94	17.5%	21.5%	49096
3-hydroxybutyrate/3-aminoisobutyrate	1.19 (d)	1.187	1.194	3.45	7.1%	4.9%	37054/49096
3-hydroxyisobutyrate	1.08 (d)	1.078	1.084	3.35	5.6%	4.8%	11805
3-hydroxyisovalerate	1.27	1.272	1.276	3.67	7.5%	4.3%	82957
3-indoxylsulfate	7.52	7.514	7.52	2.95	7.3%	8.5%	43355
4-deoxyerythronic acid	1.1	1.103	1.107	3.24	8.3%	6.3%	86347
4-deoxythreonic acid	1.24	1.239	1.244	3.48	5.5%	4.4%	86391
5-oxoproline	2.39	2.391	2.397	3.23	5.6%	7.2%	16010
Acetate	1.93	1.926	1.93	3.37	10.2%	10.2%	30089
Acetone	2.24	2.238	2.241	2.67	15.6%	19.3%	15347
Alanine	1.49	1.489	1.497	3.91	4.1%	2.2%	16977
Carnitine	3.23	3.227	3.235	4.13	12.1%	18.7%	17126
Citrate	2.54	2.514	2.575	4.91	6.6%	5.5%	30769
Creatine	3.94	3.934	3.94	5.05	4.5%	2.7%	16919
Creatinine	4.06	4.05	4.072	5.44	5.7%	3.1%	16737
Dimethylamine	2.73	2.72	2.736	4.51	4.2%	2.8%	17170
Formate	8.47	8.463	8.467	3.53	8.4%	5.2%	15740
Glucose*	5.25	5.24	5.261	3.72	2.8%	2.7%	17234
Glutamine	2.46	2.435	2.475	4.39	12.6%	11.0%	28300
Glycine	3.57	3.572	3.576	4.46	7.4%	3.8%	15428
Hippurate	7.56	7.538	7.574	4.47	2.4%	3.3%	132966
Isoleucine	1.02	1.018	1.023	2.42	9.4%	10.9%	24898
Lactate	1.33	1.329	1.336	3.75	5.9%	3.8%	24996
Leucine	0.97	0.963	0.969	2.96	6.4%	4.6%	25017
Lysine	1.74	1.715	1.756	3.80	2.3%	4.5%	25094
*N*^1^-methylnicotinamide	9.28	9.265	9.292	2.83	12.7%	10.7%	16797
*N*-acetylneuraminic acid*	2.06	2.054	2.071	4.01	4.4%	3.9%	45744
*N*-methyl-2-pyridone-5-carboxamide	6.67	6.66	6.681	3.13	9.2%	15.4%	27410
*N*-methylnicotinic acid	9.12	9.11	9.135	3.07	9.0%	7.3%	50521
*N*-methylpicolinic acid	8.71	8.698	8.725	1.70	22.0%	60.3%	69061
Pantothenic acid	0.94	0.933	0.938	3.07	6.0%	3.7%	7916
*p*-cresol sulfate	2.35	2.343	2.349	4.11	5.7%	4.2%	82914
*p*-hydroxyphenylacetate	6.87	6.871	6.878	3.23	7.8%	5.7%	18101
Proline betaine	3.3	3.301	3.308	3.83	11.0%	20.3%	35280
*Scyllo*-inositol	3.36	3.362	3.367	3.67	10.8%	9.4%	10642
Succinate	2.41	2.41	2.414	3.85	11.5%	19.6%	26806
Sucrose	5.42	5.41	5.425	3.18	3.7%	8.5%	17992
Taurine	3.43	3.419	3.45	4.67	3.2%	5.9%	15891
Trimethylamine	2.87	2.868	2.872	3.59	8.3%	7.3%	18139
Trimethylamine*-N-*oxide	3.28	3.265	3.285	5.00	2.5%	7.9%	15724
Tyrosine	6.91	6.905	6.912	3.20	8.2%	5.3%	18186
Urea	5.81	5.675	5.95	5.96	4.1%	2.7%	16199
Valine	1.04	1.039	1.044	2.93	6.7%	4.5%	27266

*Multiple overlapping resonances. Integral assigned to the most likely, abundant metabolite. **Chemical Entities of Biological Interest (ChEBI), dictionary of molecular entities available online at http://www.ebi.ac.uk/chebi/. For metabolites with chiral enantiomers, the ID without *L* or *D* specification was selected since both forms may be present in human urine.

**Table 2:** Metabolite concentrations in urine of children aged 8–9 years old sampled daily over 6 days measured by ^1^H NMR spectroscopy.

Metabolite concentration (μmol/mmol creatinine)	Daily Pool (n = 108)	Morning (n = 108)	Night (n = 108)
Creatinine (mmol/L)	6.01 (4.6–7.29)	6.12 (4.12–8.06)	5.24 (3.94–7.21)
2-hydroxyisobutyrate	6.4 (5.8–7.6)	6.8 (5.8–8.2)	6.8 (5.7–7.7)
3-hydroxyisobutyrate	16.4 (12.5–21)	15.1 (11.6–20)	18.7 (13.2–23.8)
4-deoxyerythronic acid	12.5 (10–16.6)	13.6 (10.8–19)	13 (9.6–16.4)
4-deoxythreonic acid	21.3 (17.6–26.3)	24.3 (18.8–27.8)	18.7 (14.1–23.2)
Acetate	9.1 (7.7–11.3)	8.4 (6.5–10.1)	11.4 (8.3–14.6)
Alanine	58.9 (44.9–76.1)	56 (42–67.9)	65.7 (47–87.3)
Carnitine	16.2 (8.9–24.7)	14.6 (7.3–22.2)	20.6 (11.2–31)
Citrate	449.9 (332.4–567.1)	370.3 (262.2–492)	561.4 (352.6–698.8)
Creatine	422.5 (260.8–616)	358.5 (200.4–568.4)	454.7 (306.4–663.6)
Dimethylamine	63.4 (57–76.9)	65 (57.8–74.7)	62.9 (54.5–75.7)
Formate	19.3 (13.8–26)	17.4 (12–23.8)	22.8 (14.7–30.4)
Glutamine	133.3 (112–157.8)	126.8 (102.1–145.4)	135.6 (109.8–168.5)
Glycine	117.1 (88.6–153.1)	112.4 (75.3–155.9)	125.5 (93–178.7)
Hippurate	174 (111.1–249.9)	173 (112.1–236.8)	155.7 (92.5–242.8)
Isoleucine	2 (1.6–2.3)	1.9 (1.6–2.3)	2.1 (1.6–2.6)
Lactate	38.4 (31.4–47.9)	36.3 (28.6–47.9)	39.5 (30.6–57.8)
Leucine	3.3 (2.8–3.7)	3.2 (2.8–3.8)	3.3 (2.6–4.2)
Lysine	39.4 (26.7–55)	41.1 (25–60.7)	38.6 (25.8–53.8)
*p*-cresol sulfate	47.1 (33.2–63.6)	48.1 (30.8–68.2)	47.4 (31.9–65)
*Scyllo*-inositol	12.4 (8.4–15.2)	10.7 (7.3–14.3)	12 (8.1–17.2)
Succinate	19.8 (13.4–27.8)	18.6 (9.1–25.4)	26.2 (15.5–35)
Taurine	234.6 (195.3–329.1)	209.6 (139.7–297.4)	284.6 (222.4–364.5)
Trimethylamine	4.4 (3.2–6.4)	4.9 (3.4–6.4)	4.8 (3.3–6.4)
Trimethylamine-N-oxide	111.6 (80.5–216.5)	94.3 (64.9–159.8)	110.3 (80.9–210.7)
Valine	6.5 (4.9–7.4)	6.4 (5.1–7.6)	6.7 (5.1–8.4)

To correct for variations in urine concentration, values for all metabolites were normalised to creatinine. Median (interquartile range) are presented.
