# Supplementary material for: Assessment of metabolic phenotypic variability in children’s urine using 1H NMR spectroscopy
Source: Sci Rep. 2017 Apr 19;7:46082. doi: 10.1038/srep46082 (PMC5395814; doi:10.1038/srep46082)
Supplement: Supplementary Information [file srep46082-s1.pdf]

Supplementary Information

Metabolic variability in children's urinary NMR spectroscopic based metabolic phenotypes

Léa Maitre* (1,2,3,4), Chung-Ho E. Lau (4, 5), Esther Vizcaino (4), Oliver Robinson (6), Maribel Casas (1,2,3), Alexandros P. Siskos (4, 5), Elizabeth Want (4), Toby Athersuch (4, 6), Remy Slama (7), Martine Vrijheid (1,2,3), Hector C. Keun (5), Muireann Coen* (4)

1. ISGlobal, Centre for Research in Environmental Epidemiology (CREAL) Barcelona, Spain
2. Universitat Pompeu Fabra (UPF), Barcelona, Spain
3. CIBER Epidemiología y Salud Pública (CIBERESP), Madrid, Spain
4. Computational and Systems Medicine, Department of Surgery and Cancer, Imperial College London, London, SW7 2AZ, UK
5. Division of Cancer, Department of Surgery and Cancer, Imperial College London, Institute of Reproductive and Developmental Biology (IRDB), Hammersmith Hospital, London W12 0NN
6. MRC-PHE Centre for Environment and Health, School of Public Health, Faculty of Medicine, Imperial College London, London UK
7. Inserm, Univ. Grenoble Alpes, CNRS, IAB (Institute of Advanced Biosciences), Team of Environmental Epidemiology applied to Reproduction and Respiratory Health, F-38000 Grenoble, France

SI table 1. T1 correction factor for metabolite quantification

Compounds/features	Resonance quantified	T1 (sec)
**2-hydroxyisobutyrate**	1.36 (s)	1.62
**3-hydroxyisobutyrate**	1.08 (d)	2.09
**4-deoxyerythronic acid**	1.11 (d)	2.52
**4-deoxythreonic acid**	1.24 (d)	1.12
**Acetate**	1.92 (s)	2.97
**Alanine**	1.48 (d)	1.58
**Carnitine**	3.23 (s)	1.19
**Citrate**	2.55 (d)	0.69
**Creatine**	3.94 (s)	0.96
**Creatinine**	4.06 (s)	2.07
**Dimethylamine**	2.72 (s)	3.36
**Formate**	8.46 (s)	6.03
**Glutamine**	2.45 (m)	1.28
**Glycine**	3.57 (s)	2.54
**Hippurate**	7.55 (t)	2.13
**Isoleucine**	1.01 (d)	2.06
**Lactate**	1.33 (d)	1.43
**Leucine**	0.96 (t)	1.68
**Lysine**	1.73 (m)	2.57
**p-cresol sulfate**	2.35 (s)	2.04
***Scyllo*-inositol**	3.36 (s)	5.53
**Succinate**	2.41 (s)	2.04
**Taurine**	3.44 (t)	2.14
**Trimethylamine**	2.87 (s)	1.72
**Trimethylamine oxide**	3.27 (s)	1.82
**TSP**	0(s)	2.69
**Valine**	1.05 (d)	2.28

SI Table 2 Univariate analyses of urinary metabolite differences between morning and night-time spot samples. Bolded values are significant (p-value< 0.0011, based on bonferoni correction for 44 tests) based on the Wilcox test. Fold change was calculated as the difference between morning and night concentrations concentration divided by the morning concentration. The median of the fold changes is presented across all individuals.

	Morning/Night		
	Fold change (median %)	p-values	Interquartile range
**2-hydroxyisobutyrate**	0%	5.5E-01	-0.22 - 0.19
**3-aminoisobutyrate**	-5%	7.4E-01	-0.89 - 0.51
**3-hydroxybutyrate/3-aminoisobutyrate**	-8%	9.3E-02	-0.37 - 0.14
**3-hydroxyisobutyrate**	-24%	2.6E-02	-0.43 - 0.092
**3-hydroxyisovalerate**	-5%	6.5E-01	-0.27 - 0.2
**3-indoxylsulfate**	0%	5.8E-01	-0.59 - 0.44
**4-deoxyerythronic acid**	4%	5.0E-02	-0.13 - 0.33
**4-deoxythreonic acid**	**23%**	**1.3E-06**	**0.0047 - 0.39**
**5-oxoproline**	**-11%**	**5.4E-04**	**-0.32 - 0.03**
**Acetate**	**-36%**	**3.1E-08**	**-1 - -0.013**
**Acetone**	-7%	2.3E-01	-0.95 - 0.37
**Alanine**	**-17%**	**8.0E-04**	**-0.65 - 0.091**
**Carnitine**	**-41%**	**1.2E-03**	**-1.6 - 0.27**
**Citrate**	**-52%**	**8.3E-09**	**-1.1 - -0.14**
**Creatine**	-27%	2.1E-02	-1 - 0.18
**Creatinine**	14%	9.0E-02	-0.18 - 0.33
**Dimethylamine**	3%	3.9E-01	-0.2 - 0.25
**Formate**	**-31%**	**5.3E-04**	**-0.87 - 0.14**
**Glucose**	**-10%**	**9.2E-03**	**-0.35 - 0.099**
**Glutamine**	-7%	6.3E-02	-0.33 - 0.15
**Glycine**	-12%	4.7E-02	-0.64 - 0.23
**Hippurate**	10%	5.2E-01	-0.53 - 0.36
**Isoleucine**	-11%	6.6E-02	-0.41 - 0.17
**Lactate**	-9%	3.5E-02	-0.54 - 0.16
**Leucine**	-4%	5.3E-01	-0.29 - 0.19
**Lysine**	6%	6.7E-01	-0.37 - 0.38
**N^1-methyl-nicotinamide**	**53%**	**3.2E-19**	**0.25 - 0.71**
**N-acetyl neuraminic acid**	9%	1.1E-01	-0.091 - 0.18
**N-methyl nicotinic acid**	-19%	3.4E-02	-1.5 - 0.38
**N-methyl-2-pyridone-5-carboxamide**	**23%**	**1.1E-03**	**-0.15 - 0.49**
**N-methylpicolinic acid**	-83%	1.2E-01	-Inf - 0.92
**Panthotenic acid**	**-20%**	**6.9E-05**	**-0.39 - 0.026**
**P-cresol sulfate**	1%	8.3E-01	-0.55 - 0.36
**p-hydroxyphenylacetate**	**-24%**	**2.3E-04**	**-0.9 - 0.12**
**Proline betaine**	-1%	7.8E-01	-1.7 - 0.7
***Scyllo*-inositol**	-12%	4.0E-02	-0.44 - 0.14
**Succinate**	**-41%**	**1.7E-05**	**-1.3 - 0.098**

**Sucrose**	**-148%**	**5.3E-12**	**-5.8 - -0.15**
**Taurine**	**-36%**	**3.7E-05**	**-1.2 - 0.19**
**Trimethylamine**	3%	6.1E-01	-0.11 - 0.18
**Trimethylamine oxide**	-17%	6.0E-02	-0.9 - 0.39
**Tyrosine**	-5%	1.4E-01	-0.43 - 0.17
**Urea**	-12%	5.0E-02	-0.58 - 0.2
**Valine**	-5%	2.2E-01	-0.3 - 0.16

SI Table 3 Univariate analyses of urinary metabolite differences between male and female children. Bolded values are significant (p-value< 0.0011, based on bonferoni correction for 44 tests) based on the Wilcox test. Fold change was calculated as the difference between the median male and median female concentration divided by the median male concentration. The median was calculated across all individuals.

	**Male/Female**	
	**Fold change (median %)**	**p-values**
**2-hydroxyisobutyrate**	53%	1.4E-01
**3-aminoisobutyrate**	**43%**	**6.5E-15**
**3-hydroxybutyrate/3-aminoisobutyrate**	**40%**	**5.3E-16**
**3-hydroxyisobutyrate**	**38%**	**1.6E-20**
**3-hydroxyisovalerate**	**38%**	**1.7E-07**
**3-indoxylsulfate**	**37%**	**7.3E-05**
**4-deoxyerythronic acid**	**35%**	**2.0E-08**
**4-deoxythreonic acid**	**33%**	**4.2E-08**
**5-oxoproline**	**30%**	**1.1E-05**
**Acetate**	**30%**	**6.1E-14**
**Acetone**	**28%**	**5.9E-12**
**Alanine**	**26%**	**1.7E-11**
**Carnitine**	**25%**	**1.4E-10**
**Citrate**	**24%**	**1.7E-07**
**Creatine**	**22%**	**5.8E-06**
**Creatinine**	**21%**	**3.8E-12**
**Dimethylamine**	**19%**	**7.5E-07**
**Formate**	19%	3.0E-02
**Glucose**	**18%**	**4.9E-09**
**Glutamine**	**18%**	**5.1E-04**
**Glycine**	**17%**	**6.9E-06**
**Hippurate**	17%	3.6E-02
**Isoleucine**	**17%**	**3.0E-05**
**Lactate**	**14%**	**3.9E-04**
**Leucine**	**14%**	**3.5E-08**
**Lysine**	**14%**	**1.7E-09**
**N^1-methyl-nicotinamide**	13%	5.4E-02
**N-acetyl neuraminic acid**	**12%**	**8.4E-04**
**N-methyl nicotinic acid**	**12%**	**4.3E-07**

**N-methyl-2-pyridone-5-carboxamide**	**11%**	**3.0E-03**
**N-methylpicolinic acid**	11%	9.9E-02
**Pantotenic acid**	8%	3.0E-01
**P-cresol sulfate**	7%	3.4E-01
**p-hydroxyphenylacetate**	7%	7.6E-01
**Proline betaine**	5%	6.3E-01
***Scyllo*-inositol**	5%	6.3E-01
**Succinate**	4%	8.3E-01
**Sucrose**	2%	2.1E-01
**Taurine**	**1%**	**4.1E-04**
**Trimethylamine**	-1%	9.1E-01
**Trimethylamine oxide**	-2%	7.4E-01
**Tyrosine**	-13%	5.4E-01
**Urea**	-14%	2.2E-02
**Valine**	**-20%**	**5.6E-04**

SI Table 4 Diurnal metabolic variation in urine samples (N=108 pairs first morning and night-time samples) across 6 consecutive days in 20 children aged 8-9 yrs old. The ratios represent the difference between the morning and night samples divided by the mean of the two samples. Colours are associated with the direction of the metabolite concentration difference, higher concentration in morning samples are in green, lower in red.

Day/ Child	Acetate						Acetone						Alanine						Carnitine					
	1	2	3	4	5	6	1	2	3	4	5	6	1	2	3	4	5	6	1	2	3	4	5	6
1	-0.08		-0.22	-0.01	-1.00	-0.03	-0.38		0.04	0.12	0.50	0.06	0.03		-0.06	-0.32	-0.45	0.58	-0.84		0.01	-1.40	-1.11	0.14
2	-0.54	-0.91		0.00	0.89	-0.20	-0.20	-1.41		0.72	-0.44	-0.14	0.13	0.38		-0.38	0.21	-0.03	1.03	1.60		-1.37	0.98	0.24
3	-0.28	0.31	-1.37	0.28	-0.87	0.33	-0.70	-0.49	-0.37	-0.35	0.22	0.82	-0.12	0.38	-0.25	0.29	-0.16	-0.57	-1.64	-0.47	0.92	-1.84	0.58	-1.71
4	0.17	0.31	1.11	-0.21		-0.50	0.66	0.08	0.88	-0.66		-0.17	0.06	-0.41	0.64	-0.26		0.19	0.40	1.01	0.25	-0.40		0.65
5	-0.16	-0.66	-0.54	-0.83	-0.43	-0.43	0.47	-0.52	-0.43	-0.66	-0.65	-2.00	-0.69	-0.30	0.10	-1.08	-0.30	-0.40	-0.94	0.23	-1.00	-0.73	0.03	-0.40
6		-0.03	-0.34	-0.37	-0.55	0.27		0.43	-0.50	-0.18	-0.24	0.11		0.03	-0.14	0.33	-0.47	0.17		-0.41	-0.26	-1.83	0.20	0.06
7	0.32		-0.12	-0.59	-0.88	-0.05	0.28		-1.45	-0.13	2.00	0.09	0.00		-0.37	-0.40	-1.01	-0.50	0.82		0.57	-1.44	-1.33	0.61
8	-0.01	-0.52	0.57	-0.11	-0.12	-0.45	-1.76	0.31	-1.60	0.19	0.20	0.42	1.23	-0.55	0.78	-0.70	0.37	-0.71	0.75	-1.09	1.00	-0.20	1.39	-1.32
9	-0.80	0.28	-0.86	0.77	-0.47	-1.36	-0.45	1.14	-1.21	-0.64	0.70	0.45	-0.43	0.49	0.13	0.94	-0.58	0.02	0.85	1.08	-0.89	-0.52	-0.93	-0.17
10		0.29	-0.21	-0.14	0.28	-0.10		-0.02	-0.55	-0.29	0.50	-0.06		0.52	-1.03	0.05	0.63	-0.49		-0.59	0.46	-0.22	-0.20	-0.62
11	-0.15	-0.47	-0.42	-0.77	0.05	-0.07	-0.30	-0.33	-1.64	-1.16	-0.99	-0.37	0.64	0.08	0.04	-0.40	-0.28	-0.17	0.27	0.12	-1.51	-0.69	-1.93	-0.67
12	-0.12	-0.37	0.05	0.06	-0.25	-1.03	0.44	-0.72	0.46	-0.29	0.04	0.46	0.59	0.32	-0.27	0.02	0.09	-0.27	0.90	-0.45	-1.22	0.09	0.29	0.84
13	-0.35	0.06	0.19	-0.39	-0.39	-0.77	-1.72	-0.61	0.81	-0.77	-1.16	0.74	-0.12	0.19	-0.43	-0.09	-0.15	-0.58	-0.22	-0.39	-0.14	-0.06	-0.47	-1.12
14	-0.17	-0.51		2.00	-1.11	-0.99	0.21	-0.54		0.41	2.00	-2.00	0.10	-0.36		-0.14	-0.72	-0.85	1.08	0.15		-0.96	-1.35	-0.47
15	0.17	-0.18	0.22	-0.16	-1.43	-0.33	-0.55	0.78	-0.11	0.43	0.73	0.51	0.10	-0.63	0.17	-0.11	-0.30	-0.99	-0.46	1.10	-0.70	-0.56	0.92	-0.88
16	-1.42	-0.50			-0.38	-0.61	-1.18	-0.67			1.13	-0.38	-0.87	-0.83			-0.13	-0.86	-0.20	-0.89			-0.22	-0.74
17	1.29	0.40	-0.12		-0.73	-0.25	1.26	0.59	0.53		0.23	-0.76	0.52	-0.19	-0.87		-1.14	-0.16	0.57	0.47	-0.94		-0.44	0.45
18	-0.01	-1.11	-0.22	-1.60	-1.35	-0.27	0.14	-0.80	-1.34	-1.67	0.89	-1.06	-0.44	-1.05	0.09	-0.76	-1.47	-0.32	-0.57	-0.71	0.64	-1.78	-1.35	-0.86
19		-1.15		-1.15	-0.65	-0.66		-1.54		-0.23	0.29	0.95		-0.20		-1.37	-0.15	0.05		0.40		-0.88	-1.45	-0.17
20	-0.74	-0.78	-1.00	-0.86	-0.16	-0.84	-0.92	1.09	0.73	1.19	0.54	-0.41	-0.34	-0.68	-1.25	-0.87	0.52	-0.31	-0.23	-1.46	-1.29	-0.69	0.03	-1.28

Day/ Child	Citrate						Creatine						Creatinine						Dimethylamine					
	1	2	3	4	5	6	1	2	3	4	5	6	1	2	3	4	5	6	1	2	3	4	5	6
1	-0.31		-0.14	-0.51	-0.78	0.37	-0.26		0.10	0.01	-0.06	0.39	0.29		0.40	0.01	-0.06	-0.04	0.36		0.18	-0.12	-0.08	0.26
2	-0.36	-0.43		0.18	0.18	-0.11	0.04	-0.05		0.05	-0.54	-0.53	-0.08	0.97		-0.44	-0.90	0.13	-0.11	0.69		0.39	0.34	0.11
3	-0.73	-0.32	-0.43	-0.22	-0.18	0.30	-0.03	0.89	1.15	0.21	0.85	-1.79	0.49	-0.15	1.36	0.22	0.14	-0.83	0.48	-0.05	-0.25	0.04	-0.14	-0.15

4	0.06	-0.09	0.44	-2.00		-0.73	-1.19	0.34	0.42	-0.48		0.92	-0.74	0.40	-0.79	0.08		0.34	0.09	-0.15	-0.81	0.77		-0.28
5	-0.17	-0.87	-0.24	-0.93	-0.69	-0.43	0.32	0.03	-1.14	-0.27	-0.25	-1.04	-0.17	0.41	-0.07	0.57	0.49	0.29	0.29	-0.40	-2.00	0.15	-2.00	-0.20
6		-0.49	-0.70	-0.98	-0.46	-0.49		-0.74	-0.63	0.54	-0.03	0.35		0.48	-0.11	-0.07	-0.02	0.05		0.30	0.12	0.09	0.13	-0.20
7	-0.89		-0.47	-1.03	-0.67	-0.15	-0.83		0.46	-1.09	-0.52	-0.22	-0.03		1.02	0.31	-0.03	0.18	0.07		0.11	0.18	0.00	-1.49
8	0.51	-0.90	0.38	-0.38	-0.27	-0.61	0.58	-0.92	1.12	-0.43	0.84	-0.84	-0.29	-0.10	-0.34	0.81	-0.52	0.52	-0.45	0.37	0.10	0.35	-0.29	0.02
9	0.24	-0.39	-0.01	0.63	-0.56	-1.02	-0.70	0.02	-0.89	-0.50	-0.10	0.14	-0.24	-0.27	0.22	-0.94	-0.21	0.15	-0.18	-0.09	0.18	0.06	0.03	-0.09
10		0.50	-0.39	-0.98	-0.07	-0.80		-0.06	-0.38	0.84	-0.26	0.46		-0.58	0.38	0.42	-0.55	-0.02		-0.65	0.33	0.14	-0.63	0.55
11	0.14	-0.29	-0.39	-0.91	0.23	0.10	0.47	0.18	-0.46	-0.38	-0.57	-0.02	0.07	0.45	0.24	0.66	0.02	-0.01	0.04	0.33	0.05	0.03	0.20	-0.04
12	-0.10	-0.04	-0.36	0.06	-0.34	-0.22	0.61	-0.76	0.29	-0.10	0.02	-0.19	0.13	0.67	-0.38	0.11	0.00	0.40	-0.48	-0.27	0.33	0.21	-0.15	-0.97
13	-0.05	-0.57	-0.38	-0.58	-0.46	-0.55	-0.60	0.25	0.16	0.07	0.60	-1.06	0.63	-0.13	-0.47	0.02	0.39	0.12	-0.03	0.06	0.00	-0.04	-1.15	0.69
14	-0.33	-0.22		-0.40	-0.81	-1.02	0.57	0.45		0.12	-0.94	-1.32	0.03	0.46		-0.55	0.29	0.66	-0.03	-1.40		0.83	0.47	-0.12
15	-0.16	-0.70	-0.15	0.84	-0.62	-0.72	-0.18	-0.13	-0.33	-1.44	1.16	-0.73	0.58	-0.37	-0.22	-0.43	-0.13	-0.16	-0.70	0.24	-0.49	0.61	-0.71	0.56
16	-1.06	-1.28			-0.45	-0.18	-0.01	-0.88			-0.02	-0.59	0.83	0.40			-0.16	0.51	-0.07	0.37			-0.37	-0.05
17	0.71	-0.04	-0.03		-0.78	-0.34	-0.29	-0.09	-0.13		0.71	-0.78	-1.20	-0.72	-0.90		-0.29	0.28	-0.15	0.11	0.42		-0.04	0.34
18	-0.31	-0.41	-0.38	-1.09	-0.89	-0.78	0.12	-0.93	-0.47	-1.31	-0.94	0.19	0.08	1.10	1.04	1.21	0.49	0.27	-0.33	-0.18	0.09	-0.12	0.50	-0.19
19		-0.61		-0.90	-0.53	-0.39		-0.25		-1.60	-1.25	0.54		-0.62		0.66	0.05	-0.06		0.19		-0.58	0.57	-0.89
20	-0.34	-0.46	-0.77	-0.94	-0.07	-0.18	-0.67	-0.94	-0.87	-0.99	-0.56	0.02	0.79	-0.06	-0.05	0.08	-0.70	0.37	0.66	1.70	0.09	0.40	0.62	-0.09

	Formate						Glucose						Glutamine						Glycine					
1	0.16		-0.02	-0.25	-0.82	0.34	0.07		-0.30	0.06	-0.65	0.32	0.04		0.29	-0.31	-0.16	0.64	0.25		0.17	-0.45	-0.30	0.59
2	0.23	-0.79		-0.03	0.94	-0.10	0.03	0.17		-0.34	0.08	-0.02	0.19	0.09		0.00	0.45	0.23	0.17	0.33		0.01	0.25	0.37
3	-0.51	-0.08	-0.88	0.32	-0.90	-0.10	0.06	-0.39	-0.27	0.00	-0.06	-0.50	0.26	0.29	-0.93	0.79	-0.75	-0.22	-0.06	0.31	-0.36	0.38	-0.18	-0.49
4	0.60	-0.01	1.11	-0.02		0.66	0.36	-0.08	0.18	0.09		-0.23	0.51	-0.02	0.50	-0.28		0.15	0.56	-0.15	1.03	-0.36		-0.09
5	-0.32	-0.55	0.35	-1.50	-0.01	-0.19	0.24	-0.53	0.02	-0.63	-0.44	-0.02	-0.10	-0.05	0.04	-0.28	-0.47	-0.11	-0.41	0.05	-0.05	-0.93	-0.23	-0.30
6		0.17	-0.09	0.05	-0.47	0.30		0.06	-0.06	0.01	-0.13	-0.05		0.19	-0.37	-0.11	-0.32	-0.24		0.13	-0.32	0.16	-0.50	0.37
7	-0.08		-0.50	0.02	-0.63	0.23	0.55		-0.15	0.17	-0.32	-0.30	0.04		0.09	0.23	-0.23	-0.02	0.19		-0.41	-0.32	-0.69	0.37
8	1.14	-0.79	0.57	-0.56	0.27	-1.10	-0.08	-0.07	-0.26	0.38	-0.16	-0.08	0.20	-0.62	0.66	-0.20	0.32	-0.08	1.05	-0.48	1.14	-0.49	0.45	-0.90
9	-0.46	0.19	-0.31	0.91	-0.19	-0.63	0.09	0.01	-0.01	0.26	0.40	-0.39	0.13	0.12	-0.11	0.46	0.02	0.20	0.09	0.07	-0.10	0.75	-0.35	0.19
10		0.73	-0.89	0.00	0.45	-0.56		0.36	-0.35	-0.04	0.16	-0.08		0.23	-0.52	-0.28	0.24	-0.10		0.70	-0.64	0.12	0.73	-0.31
11	-0.14	-0.53	-0.74	-1.07	-0.45	-1.08	0.25	0.34	0.03	-0.09	0.28	-0.21	0.22	-0.13	0.06	-1.20	-0.21	0.10	0.37	0.04	-0.26	-0.72	0.48	-0.03

12	-0.17	-0.08	-0.58	0.02	-0.36	-0.60	-0.19	-0.37	0.19	0.14	0.01	-0.21	0.37	-0.33	-0.30	-0.06	-0.17	0.22	0.54	0.30	-0.46	0.33	-0.22	-0.03
13	-0.29	0.18	-0.08	0.16	-0.32	-0.80	-0.45	0.43	0.41	-0.10	-0.52	0.13	-0.20	-0.14	0.10	-0.04	0.29	-0.01	-0.21	0.04	0.08	-0.27	-0.62	-0.74
14	0.15	1.30		0.31	-1.29	-1.21	-0.43	-0.27		-0.23	-0.98	-0.37	0.20	-0.40		0.45	-0.67	-0.94	0.20	-0.42		0.35	-0.81	-0.82
15	0.15	-0.45	0.35	-0.44	-0.90	-0.51	-0.05	-0.50	-0.06	0.30	-0.19	0.00	-0.16	0.03	-0.07	0.09	-0.04	-0.18	0.40	0.17	0.17	-0.55	0.38	-1.14
16	-0.90	-1.19			-2.00	-1.29	-0.16	-0.22			-0.46	-0.33	-1.05	-0.51			-0.11	-0.50	-1.11	-0.59			-0.19	-0.79
17	1.00	0.09	-0.20		-1.01	-0.09	0.01	0.22	-0.04		-0.90	0.16	0.89	-0.01	-0.51		-0.99	0.22	1.00	0.31	-0.80		-1.05	0.13
18	-0.07	-0.53	0.09	-1.07	-1.55	-0.34	-0.10	-0.74	0.19	0.04	-0.64	-0.40	-0.34	-0.27	-0.04	-1.25	-0.78	-0.27	0.16	-0.92	-0.28	-1.04	-1.14	-0.64
19		0.14		-1.64	-0.09	-0.59		-0.01		-0.63	0.19	-0.01		-0.21		-0.70	-0.30	0.11		0.01		-1.19	-0.27	0.26
20	0.24	-1.03	-1.55	-0.59	0.03	-0.34	0.35	-0.05	-0.40	-0.39	-0.07	0.28	-0.19	-0.09	-0.41	-0.24	0.16	-0.53	-0.07	-0.49	-0.91	-1.04	0.54	-0.78
	**Hippurate**						**N-methylpicolinic acid**						**Isoleucine**						**Lactate**					
1	-0.23		0.41	0.15	0.28	-0.61	1.63			-0.60	-0.02	-1.71	-0.13		0.09	-0.29	-0.33	0.63	0.32		0.20	-0.40	-0.12	0.56
2	0.62	0.40		0.66	0.51	-0.26	0.32	1.48		1.71	2.00	-2.00	0.51	-0.88		0.15	0.41	0.35	0.14	-0.14		0.23	0.35	0.22
3	-0.44	1.32	-0.75	-0.36	0.04	0.38	1.09	-2.00	-2.00	-2.00	-2.00	2.00	-0.01	0.82	0.18	0.43	-0.19	0.16	-0.15	0.14	-0.18	0.15	0.05	-0.22
4	0.34	-0.35	-0.07	0.33		-0.74	N	2.00	-1.99	1.78		-0.99	0.40	0.09	0.48	0.19		0.08	0.64	0.05	1.05	-0.42		0.15
5	0.95	-0.31	0.06	0.53	-0.49	0.74	0.99	-2.00	-2.00	-1.56	2.00	-2.00	0.23	-0.18	-0.39	-0.37	-0.43	-0.19	-0.16	-0.05	-0.12	-0.76	-0.35	-0.23
6		-0.05	-0.37	-0.39	0.16	-0.08		-2.00	-2.00		-0.61	-2.00		0.35	-0.28	0.06	0.18	0.19		0.07	-0.10	0.14	-0.37	0.20
7	-0.60		1.16	0.76	0.59	-0.67	2.00		2.00	-2.00	0.77	-1.98	0.06		-0.19	-0.57	-0.23	-0.26	-0.04		-0.41	-0.20	-0.59	0.26
8	-0.21	-0.48	0.40	-1.32	1.71	-1.58	-2.00	1.34	-0.54	2.00	1.95	1.43	-0.84	0.20	0.27	0.35	0.49	-1.15	0.57	-0.36	0.89	-0.47	-0.05	-0.71
9	-0.99	0.18	-0.27	-0.16	1.34	-1.22	1.23		2.00	1.12	-0.65	2.00	-0.42	0.57	-0.62	-0.42	0.17	-0.84	-0.05	0.55	-0.36	1.10	-0.44	0.50
10		1.70	0.32	-0.32	-0.31	0.62		2.00	-2.00		2.00			0.25	0.03	0.18	0.17	0.18		0.64	-0.65	0.10	0.65	-0.33
11	-1.23	1.39	-0.38	0.33	0.86	-0.05	2.00	2.00	-2.00		-2.00	-2.00	-0.12	-0.11	-0.54	-0.47	-0.50	0.16	0.50	0.10	-0.10	-0.94	0.04	0.16
12	-0.06	-0.45	0.38	0.55	-0.29	-0.41	-2.00		-2.00	-1.52	-2.00	-0.76	0.45	-0.48	-0.15	0.00	0.55	-0.19	0.49	0.14	-0.33	0.14	0.10	-0.09
13	1.01	-0.65	-1.10	-0.08	-1.08	1.21	-2.00	1.03	0.64	-1.51	-1.94	1.88	-0.44	0.31	0.12	0.19	-0.51	-0.31	-0.30	0.39	0.20	-0.55	0.20	-0.50
14	0.00	-0.41		-0.47	0.06	-0.43	-2.00	-1.97		1.04		-1.96	0.07	-0.57		0.10	-0.28	-0.83	0.31	-0.43		-0.07	-0.82	-0.88
15	-0.37	-0.25	-0.51	0.25	-1.32	0.00	-2.00	0.55	2.00	2.00	-2.00	1.67	-0.15	-0.30	-0.54	-0.74	-0.30	-0.02	0.05	0.08	0.09	-0.58	0.57	-0.80
16	0.08	0.67			0.03	-0.35	0.15	-0.90			-2.00		-0.07	-0.50			-0.09	-0.16	-0.61	-0.76			-0.06	-0.58
17	-0.60	0.16	0.19		-1.66	0.97	-2.00	2.00	0.31		-1.75	-2.00	0.51	0.61	-0.07		-0.49	0.08	0.73	0.24	-0.53		-0.61	-0.29
18	-0.44	0.19	-0.74	1.36	-0.19	0.44	2.00	0.66		-0.30	2.00		0.07	-0.50	0.25	-0.39	-0.13	0.25	-0.14	-0.98	-0.25	-0.60	-1.23	-0.39
19		0.19		-0.44	0.17	0.90		-2.00		-2.00	1.58	-1.81		0.71		-0.59	0.08	0.14		-0.81		-1.04	-0.29	-0.02

20	0.44	0.79	0.00	1.59	-0.59	0.62	-1.74	N	2.00	N	2.00	-0.71	-0.21	0.07	-0.15	-0.02	0.18	-0.38	-0.45	-0.28	-0.72	-0.85	0.42	-0.39

	Leucine					
1	0.01		0.08	-0.28	0.24	0.40
2	0.18	0.14		-0.10	0.30	0.32
3	0.20	0.28	0.12	1.11	-0.77	-0.55
4	0.56	-0.02	0.64	0.00		-0.06
5	0.12	-0.28	0.08	-0.35	-0.19	-0.01
6		-0.01	-0.17	0.19	-0.06	0.11
7	0.13		-0.15	0.24	-0.34	0.46
8	0.20	-0.32	0.57	0.00	0.40	-0.55
9	-0.35	0.43	-0.28	-0.19	0.25	0.09
10		-0.18	-0.25	0.26	0.32	-0.03
11	0.16	0.27	-0.20	-0.57	-0.30	0.29
12	0.52	-0.09	-0.08	0.12	0.24	-0.23
13	-0.06	0.18	0.35	0.35	0.06	-0.23
14	0.10	-0.19		0.20	-0.41	-0.13
15	0.07	0.17	-0.12	-0.29	0.45	-0.24
16	-0.92	-0.26			-0.11	-0.29
17	0.12	-0.01	-0.23		-0.36	-0.53
18	0.11	-0.58	-0.38	-0.43	-0.52	-0.27
19		0.28		-0.69	-0.17	0.34
20	0.27	-0.24	-0.44	-0.48	0.34	-0.26

	Lysine					
1	0.64		0.53	-0.14	0.44	1.16
2	0.12	0.10		0.07	0.17	0.46
3	-0.73	0.66	0.27	0.19	0.96	-0.17
4	0.61	-0.12	0.62	-0.11		-0.87
5	0.07	-0.29	-0.70	0.30	0.07	0.08
6		0.13	-0.25	0.47	-0.08	0.22
7	0.03		-0.60	-0.01	-0.82	0.73
8	0.93	-0.67	0.90	0.05	-0.18	0.42
9	-0.10	0.51	0.05	1.22	-0.17	0.50
10		1.07	-1.08	0.15	0.49	-0.59
11	0.67	0.19	-0.26	-1.02	0.31	0.06
12	0.96	0.64	-0.30	-0.72	0.62	0.52
13	-0.32	0.44	0.46	0.04	0.48	-0.26
14	0.75	-0.23		0.41	-0.63	-0.93
15	0.27	0.37	0.11	-0.73	0.75	-0.63
16	-0.25	-0.44			0.55	-0.36
17	0.56	0.31	-0.31		-0.37	0.11
18	0.40	-0.20	-0.51	-0.80	-0.66	-0.31
19		0.06		-1.10	0.14	-0.14
20	-0.56	-0.44	-0.59	-0.22	0.76	-0.39

	N-acetyl neuraminic acid					
1	0.15		0.01	-0.04	0.08	0.16
2	0.21	-0.02		-0.48	0.09	0.03
3	-0.42	-0.56	-0.59	0.02	-0.11	-0.17
4	0.09	-0.10	0.11	0.31		0.20
5	0.25	0.12	-0.08	-0.09	0.14	-0.07
6		0.25	0.23	0.07	0.37	0.00
7	0.70		0.11	-0.45	0.35	-0.53
8	-0.34	0.10	0.17	0.18	-0.02	0.14
9	-0.29	0.32	0.04	0.00	0.12	0.14
10		0.11	-0.09	0.14	-0.01	0.26
11	0.27	0.54	0.00	0.25	0.12	0.03
12	-0.04	-0.53	0.28	-0.08	0.20	0.12
13	0.09	0.02	0.06	0.12	-0.17	0.40
14	-0.12	-0.31		0.01	0.35	0.13
15	-0.18	-0.02	0.01	0.77	-0.10	-0.33
16	-0.03	0.23			-0.20	0.31
17	-0.26	-0.35	0.67		-0.66	-0.03
18	-0.36	-0.49	0.12	0.02	0.09	-0.26
19		0.33		-0.13	0.32	0.06
20	0.42	0.27	0.17	0.24	0.53	0.34

	N-methyl-2-pyridone-5-carboxamide					
1	0.29		1.68	0.38	-0.41	0.60
2	-0.01	0.48		1.28	0.65	0.41
3	-0.40	0.71	0.63	-0.08	-0.36	0.70
4	0.64	0.42	0.74	0.20		0.26
5	0.66	-0.52	0.60	0.12	-0.33	1.09
6		-0.24	0.52	0.74	0.20	0.03
7	N		0.35	0.46	0.48	1.02
8	0.39	-0.08	0.53	1.35	0.72	-0.10
9	-2.00	-0.33	-0.02	0.41	0.97	0.24
10		0.40	-0.01	-0.48	-0.58	-0.60
11	0.28	0.54	-0.27	-0.11	0.66	-0.04
12	0.28	-0.42	0.71	0.33	0.28	-0.23
13	-1.11	0.44	1.35	-0.67	0.31	0.55
14	0.82	-0.51		-2.00	0.37	1.73
15	-0.37	1.17	-1.27	-0.48	0.11	0.88
16	1.17	0.02			2.00	0.12
17	1.26	0.38	0.04		1.01	-0.17
18	0.99	0.54	-1.01	-0.23	1.35	-0.22
19		0.72		0.48	0.63	0.31
20	-1.46	0.15	0.13	0.01	-1.81	0.00

	N-Methyl nicotinic acid					
1	0.10		0.06	-0.26	-0.61	-0.08
2	0.03	0.46		0.48	-1.67	-0.07
3	0.82	-0.94	-0.81	1.00	-0.05	-1.43
4	0.95	-0.32	0.79	0.17		-1.12
5	-0.82	-0.15	-0.59	-0.54	-0.06	0.61
6		-0.71	0.42	0.38	-0.95	-0.44

	N^1-Methyl-nicotimide					
1	0.59		0.67	1.41	0.67	-0.02
2	0.91	-0.03		1.19	1.29	0.83
3	0.64	0.81	0.94	0.25	0.84	0.66
4	0.50	0.61	0.73	0.24		0.56
5	1.07	0.47	-0.19	0.77	0.09	1.26
6		0.13	0.21	0.58	0.82	0.43

	P-cresol sulfate					
1	1.04		-0.37	0.95	-1.05	1.32
2	-0.31	0.32		0.02	0.56	-0.19
3	-0.32	-0.15	-0.32	0.53	-0.30	0.44
4	0.25	-0.09	-0.43	0.97		-0.85
5	-0.56	-0.68	0.84	-0.84	-0.47	0.59
6		0.49	0.40	-0.69	0.21	-0.32

	p-hydroxyphenylacetate					
1	0.07		0.14	0.47	-0.40	0.28
2	0.79	1.24		-0.26	-0.77	-1.05
3	0.19	-0.43	0.44	-0.17	-0.73	-0.60
4	0.80	-1.24	-1.41	0.56		-0.44
5	-0.20	-0.90	-0.71	-0.11	-0.22	-0.09
6		-0.39	-0.55	-0.12	0.05	-0.74

7	-1.69		-1.59	1.25	-0.28	-1.17	0.49		0.49	0.17	1.29	0.60	-0.61		0.09	-0.43	-0.64	-1.18	-0.02		-0.11	0.13	-0.83	-0.58
8	-0.53	-0.84	-1.14	-0.43	-1.11	0.16	-0.08	0.19	0.51	1.69	-0.83	-0.26	0.57	0.27	1.15	-0.16	1.83	-0.40	0.14	-0.99	0.01	0.30	0.22	-0.88
9	-0.37	-0.40	0.69	1.91	0.99	-0.39	0.56	0.23	0.90	1.01	0.80	0.69	-0.78	-0.72	0.77	-0.71	1.59	-0.43	-1.29	0.98	-0.62	-1.43	0.25	-0.30
10		1.56	-1.37	-1.30	1.34	-1.23		1.19	1.14	0.14	0.22	-0.51		0.30	0.36	0.37	-0.29	0.37		0.40	-0.12	0.08	-0.25	-0.18
11	-0.88	1.00	-0.17	-0.95	0.31	-0.45	0.70	0.78	0.79	0.82	1.31	0.86	-0.36	0.61	-0.42	0.16	0.08	0.43	0.32	0.35	0.61	0.09	0.14	-0.55
12	-1.35	-1.26	-0.96	0.88	0.75	-0.92	0.60	-0.19	1.41	0.72	0.91	1.70	-1.05	-0.41	0.43	-1.21	0.05	-0.14	0.17	-1.05	0.39	-0.62	0.14	-0.03
13	0.01	-0.83	1.00	-0.22	-1.44	0.75	0.30	1.20	1.30	0.63	1.16	1.42	-0.24	-0.28	0.13	0.13	-0.26	0.88	-0.64	-0.05	0.03	-0.23	-0.60	0.42
14	-0.08	-1.19		-0.85	0.73	-0.19	1.31	0.82		0.48	1.10	1.43	-0.05	-0.44		-0.35	-0.27	0.19	-0.41	-0.27		-0.24	-1.42	0.84
15	0.40	-0.06	-0.75	-0.42	-0.52	-0.17	-0.80	0.04	0.39	0.23	1.15	1.36	-0.14	0.14	-0.04	0.82	0.05	-0.99	-0.52	-0.21	-0.87	-0.11	-1.50	-0.01
16	-0.40	-1.22			0.75	-0.53	0.82	-0.18			1.22	0.89	-0.13	0.36			0.58	-0.68	-1.35	-0.13			-0.03	-1.04
17	-1.67	0.62	0.44		-0.46	0.08	1.69	0.77	1.34		1.89	1.09	-0.33	0.62	-0.02		0.01	0.46	-0.33	0.21	0.11		-0.34	0.01
18	1.12	-1.36	-0.38	-1.20	0.97	0.58	0.55	0.74	-0.04	1.51	1.95	0.81	-0.02	0.03	-0.69	0.49	-0.82	1.65	-0.63	-0.57	-0.43	-0.19	-1.05	-0.89
19		-0.24		-1.50	1.25	-0.07		0.35		0.41	-0.12	0.65		-0.49		0.91	0.00	-0.48		-0.24		-0.99	0.45	-0.59
20	0.45	-0.47	0.79	-0.45	1.44	0.57	-0.54	0.39	1.29	-0.43	-0.06	0.85	-0.74	-0.50	1.10	0.64	1.63	-1.63	0.08	-0.40	-0.74	0.34	-0.02	-0.73
	**Pantotenic acid**						**Proline betaine**						**5-oxoproline**						**Scyllo-inositol**					
1	0.19		0.01	-0.05	-0.09	-0.22	-1.29		-1.30	1.29	0.37	-1.23	-0.11		0.07	-0.05	-0.56	0.34	-0.20		2.00	-2.00	-2.00	-2.00
2	-0.09	-0.18		0.13	0.04	0.03	-0.94	-0.91		0.27	-0.25	-1.24	0.06	-0.23		-0.14	0.46	-0.04	-0.01	-0.21		0.03	-0.01	-0.13
3	-0.22	-0.11	-0.46	0.01	-0.29	-0.18	0.00	2.00	0.97	0.42	-2.00	-1.95	-0.09	0.07	-0.56	0.17	-0.31	-0.26	0.08	-0.17	0.54	0.14	-0.23	0.71
4	0.01	-0.17	0.47	-0.20		0.03	0.10	-1.28	-0.85	-0.40		-1.05	0.50	0.03	0.22	0.04		-0.40	0.42	-0.80	0.07	0.13		-0.32
5	-0.08	-0.48	-0.38	-0.52	-0.58	-0.01	-0.40	0.84	-1.42	-1.12	1.08	-1.33	-0.30	-0.66	-0.17	-0.27	-0.52	-0.08	-0.03	0.03	-0.65	-0.65	0.22	-0.42
6		0.03	-0.39	-0.17	0.01	-0.13		-2.00	-2.00	1.26	2.00	0.97		0.00	-0.05	-0.15	0.04	-0.18		0.26	-0.03	-0.07	0.42	-0.02
7	-0.21		-0.48	-0.03	-0.27	-0.15	-0.43		2.00	-0.42	-1.73	1.32	0.31		-0.25	0.06	-0.62	-0.24	-1.26		-0.72	-0.30	-0.38	-0.09
8	-0.10	-0.01	0.05	-0.43	0.49	-0.73	-0.01	-0.22	0.30	-0.07	-0.37	-0.72	-0.49	-0.13	-0.17	0.00	-0.28	-0.46	-0.07	-0.18	0.15	0.94	-0.21	-0.60
9	-0.39	-0.01	-0.38	-0.56	-0.27	-0.14	-0.34	1.55	-1.58	1.27	1.79	1.94	-0.30	-0.08	0.32	0.32	-0.10	-0.27	-0.21	0.16	-0.07	-0.25	-2.00	-0.25
10		0.47	-0.35	0.03	0.12	0.04		0.82	0.41	1.56	-0.79	-0.19		0.29	-0.03	-0.08	-0.08	0.07		0.37	-0.35	-0.17	0.03	0.15
11	-0.30	-0.44	0.48	-0.41	0.33	-0.04	0.73	-0.88	-0.02	-0.92	1.08	-0.82	-0.08	0.07	-0.28	-0.11	0.37	-0.34	-0.94	-0.26	0.27	-0.11	-0.33	0.15
12	0.31	-0.09	-0.01	0.11	-0.06	0.16	-2.00	2.00	-0.66	-1.19	1.88	-0.29	0.08	-0.53	0.08	0.13	-0.15	-0.25	-0.03	2.00	-0.04	0.03	0.37	0.10
13	-0.42	-0.01	0.02	-0.29	0.19	-0.45	0.69	-2.00	1.87	0.29	0.86	-1.05	-0.08	-0.22	0.06	-2.00	-0.37	-0.11	-0.20	0.42	0.18	0.55	-0.35	0.18
14	-0.28	-0.56		0.08	-0.24	-0.61	0.21	-0.47		0.45	1.14	-1.53	-0.27	-0.32		0.43	-0.12	-0.09	-0.13	-0.42		0.21	-0.26	-0.06

15	-0.47	0.12	0.23	0.43	-0.19	-0.49	2.00	0.39	-2.00	2.00	2.00	-0.55	-0.07	-0.05	-0.98	0.21	-0.10	0.01	0.03	-2.00	-2.00	-0.62	0.71	-1.11
16	-0.16	0.08			-0.02	-0.14	-2.00	0.66			2.00	-1.83	-0.56	-0.20			-0.34	-0.30	-0.78	-0.20			-0.11	-0.60
17	0.40	-0.16	0.02		-0.63	-0.23	0.21	1.60	N		0.18	0.62	0.57	-0.04	-0.27		-0.99	-0.01	0.61	0.21	-2.00		2.00	-0.11
18	0.07	-0.68	-0.18	-0.71	-0.32	-0.23	0.81	-0.21	2.00	0.09	1.22	-0.51	-0.19	-0.33	-0.21	-0.47	-0.13	-0.16	-1.37	-0.28	-0.39	0.45	-2.00	-0.71
19		0.24		-0.40	-0.30	0.45		2.00		-2.00	2.00	-2.00		0.01		-0.43	0.01	0.07		0.47		-0.47	-2.00	-0.23
20	-0.23	0.02	-0.47	-0.79	-0.04	-0.23	1.28	0.63	-0.04	-2.00	0.82	2.00	-0.04	0.32	-0.21	-0.33	-0.05	-0.10	0.09	0.36	-0.29	-0.14	0.61	-0.22

	Succinate						Sucrose						Taurine						Trimethylamine					
1	0.24		0.07	-0.12	-0.91	0.58	-1.30		-1.89	-0.81	-1.63	0.14	-0.59		-0.47	-0.51	0.40	0.74	-0.20		-0.02	0.35	-0.22	0.51
2	N	-2.00		-0.15	0.69	-0.23	-1.81	0.16		-1.91	-0.05	-0.68	-0.16	-0.77		-0.44	-0.49	-0.80	0.03	-0.06		-0.03	-0.21	0.26
3	-0.48	-0.37	-1.71	-0.16	-2.00	0.10	-1.06	-1.50	-0.52	-0.99	0.08	-0.71	-0.47	0.34	-1.39	1.19	-0.65	-0.88	0.20	-0.03	-0.05	0.30	-0.02	-0.20
4	0.33	0.04	2.00	0.21		-0.80	-1.75	0.14	-0.60	-1.52		2.00	-0.16	-1.11	-0.33	0.27		-0.54	0.50	-0.18	0.07	0.23		-0.40
5	-0.27	-0.71	-0.91	-2.00	-2.00	-2.00	-1.71	-1.97	-1.35	-1.07	-0.76	-1.49	0.57	0.68	0.19	-1.64	-1.61	-1.21	0.42	-0.07	-0.06	-0.10	-0.03	0.15
6		0.92	-0.25	-0.67	0.69	-0.29		0.38	-0.82	-1.14	-1.96	-0.04		-0.32	-0.34	0.12	-0.11	0.16		0.08	0.17	0.34	0.02	0.25
7	-0.27		-0.56	0.18	-1.15	0.68	-0.61		0.63	-2.00	-0.52	-1.66	-2.00		0.09	-1.71	-0.95	-1.33	0.09		-0.17	0.18	0.31	-0.73
8	-0.41	-0.26	0.76	0.68	-0.68	-0.41	0.05	-1.86	-0.53	-0.70	-0.25	0.54	0.35	-0.57	0.42	-0.38	-0.22	2.00	-0.09	0.32	0.08	0.01	-0.08	-0.44
9	-0.73	-0.27	-0.45	0.97	-0.83	-1.24	-2.00	0.13	0.02	-1.18	-2.00	-0.76	-0.41	-0.11	-0.33	-0.05	-0.14	0.41	-0.06	0.07	-0.17	0.42	-0.08	-0.20
10		0.56	0.21	0.40	0.88	-0.18		-1.68	-1.24	-1.30	-0.31	-2.00		-0.83	-0.28	0.28	2.00	-0.32		0.55	-0.31	0.24	-0.12	0.00
11	-0.61	-2.00	-2.00	-0.74	0.55	-0.42	1.13	-0.24	1.19	0.07	-0.46	-0.07	0.56	-0.99	-0.50	-0.22	-0.30	1.30	0.06	0.21	-0.02	-0.03	0.13	-0.17
12	-0.55	-0.56	-0.16	0.88	-0.33	-1.16	-0.99	-0.16	-1.37	0.36	-0.80	-2.00	0.32	-0.45	0.45	0.09	-0.52	-0.25	0.22	-0.20	0.25	-0.05	0.10	0.09
13	-2.00	-0.58	0.51	-0.28	-0.79	-0.50	-0.32	0.58	-1.83	-0.47	0.09	-0.09	-1.19	0.13	-0.03	-0.21	-0.75	-0.35	0.06	0.11	0.07	0.20	-0.40	0.05
14	0.09	-0.50		1.66	-0.91	-1.76	-1.20	-0.82		-1.09	-0.36	-0.87	1.91	-1.65		0.74	-0.89	-1.48	-0.04	-0.88		0.49	-0.07	0.14
15	0.12	-0.50	-0.21	0.19	-1.19	-0.18	-0.36	-0.90	-0.24	1.16	-1.97	-0.26	-0.46	-0.09	-0.52	-0.50	0.45	0.90	-0.14	0.34	-0.28	-0.05	0.13	0.11
16	-2.00	-0.91			-1.53	-0.51	-0.79	-0.80			-0.44	-0.39	-0.42	-0.97			0.09	-0.26	-0.08	0.20			0.01	0.00
17	1.64	-0.28	0.10		-0.70	-2.00	-1.33	0.65	-1.45		-1.69	-0.22	0.54	-0.77	0.69		1.05	0.22	0.15	0.13	0.54		-0.18	0.29
18	-0.02	-0.71	-2.00	-2.00	-0.21	-0.39	-1.00	-1.98	-0.63	-0.51	-1.72	-0.58	-0.04	-0.75	-0.82	-1.23	-1.14	0.41	0.53	-0.30	-0.40	-0.76	0.01	0.32
19		-1.17		-0.68	-0.36	0.11		0.97		-1.88	-1.37	0.36		-1.16		-1.16	-0.72	-0.11		0.62		-0.67	0.48	-0.19
20	-0.61	-0.46	-0.54	2.00	-0.07	-1.21	1.21	-2.00	-0.85	-1.81	-1.81	0.44		-0.16	-0.50	0.40	-0.18	-0.74	-0.10	-0.07	0.00	-0.47	0.57	0.23

	Trimethylamine oxide						Tyrosine						Urea						Valine					
1	1.53		1.36	0.51	-1.01	0.93	-0.06		-0.34	-0.04	-0.14	0.26	-0.31		-0.35	-0.25	-0.18	0.04	0.02		-0.06	-0.20	0.11	0.07

2	-0.20	1.71		1.25	0.19	0.48	0.46	-0.02		-0.40	0.70	0.44	0.12	-1.08		0.63	1.00	-0.26	0.28	-0.26		-0.01	0.55	0.31
3	0.31	-0.65	-0.80	0.44	-0.14	-0.61	0.01	-0.16	-0.48	0.19	0.05	-0.65	-0.26	0.10	-1.13	-0.05	-0.17	0.66	0.08	0.30	-0.29	0.38	0.03	-0.11
4	-0.12	-0.24	-1.29	1.06		-0.50	0.40	-0.58	0.30	-0.28		0.34	0.67	0.10	0.87	-0.28		-0.26	0.53	-0.12	0.63	-0.27		0.01
5	1.58	-2.00	0.16	-0.19	-0.23	-1.65	0.14	-0.13	0.19	-0.23	-0.51	-0.28	0.48	-0.23	0.26	-0.50	-0.62	-0.26	0.15	0.12	0.09	-0.56	-0.56	-0.05
6		0.47	0.59	0.83	0.08	-0.45		0.10	0.22	-0.05	-0.02	-0.03		-0.16	-0.12	0.13	-0.03	0.03		0.18	-0.15	0.05	-0.02	0.23
7	1.18		1.38	-0.04	-1.04	-1.89	0.28		-0.77	0.30	-0.33	0.63	-0.05		-0.84	-0.23	-0.37	0.12	-0.29		-0.19	0.25	-0.39	0.57
8	0.26	0.47	0.79	0.30	0.04	-1.05	0.11	0.05	0.76	-1.07	0.74	-0.97	0.29	0.01	0.51	-0.36	0.43	-0.66	0.10	-0.21	0.45	0.05	0.11	-0.65
9	-1.37	0.69	0.01	0.04	1.84	-0.12	-0.30	0.40	-0.34	-0.06	-0.18	-0.06	0.05	0.58	-0.56	0.78	0.35	-0.38	-0.11	0.53	-0.25	0.31	0.11	0.22
10		-0.77	0.24	0.18	-1.76	0.60		0.71	-0.72	-0.01	0.69	0.00		0.69	-0.51	-0.30	0.24	0.14		0.59	-0.50	0.08	0.43	-0.02
11	0.07	1.21	-0.16	-0.03	0.32	-0.68	-0.15	0.54	-0.34	-0.30	-0.34	0.05	0.30	-0.79	0.04	-0.66	-0.05	0.27	-0.16	-0.02	-0.46	-0.71	-0.26	0.08
12	-0.41	0.35	-0.33	-0.48	-1.29	0.51	0.19	0.02	-0.16	0.56	-0.05	-0.21	0.03	-0.60	0.32	-0.14	0.40	-0.61	0.34	-0.30	-0.07	0.19	0.21	-0.20
13	-1.56	1.14	0.09	-0.19	-1.74	1.52	-0.57	0.01	0.23	-0.10	-0.07	-0.08	-0.82	0.18	0.52	-0.15	-0.64	-0.65	-0.29	0.07	0.35	0.05	-0.09	0.07
14	0.47	-1.95		1.71	0.04	-1.43	-0.20	-0.53		0.30	-0.89	-0.49	0.32	-0.40		0.56	-0.98	-1.00	0.01	-0.30		0.17	-0.16	-0.62
15	-1.60	0.65	-1.38	-0.17	-1.39	0.52	-0.13	0.25	-0.02	-0.44	0.17	-0.41	-0.44	0.18	0.28	-0.17	0.22	-0.44	0.01	0.07	0.09	-0.27	0.34	-0.30
16	-0.10	0.39			-0.47	-0.56	-0.60	0.02			-0.45	-0.58	-0.55	-0.49			0.19	-0.38	-0.50	-0.56			0.01	-0.37
17	-0.33	-0.11	1.74		0.21	0.21	0.55	-0.39	-0.26		-0.43	0.13	0.95	0.19	0.55		0.15	-0.48	0.20	-0.20	-0.19		-0.40	-0.22
18	-0.93	-0.48	0.05	-0.60	-0.51	-0.51	-0.24	-0.78	-0.48	-0.43	-0.06	-0.53	0.21	-0.86	-0.72	-1.28	-0.38	-0.08	0.29	-0.32	-0.15	-0.47	-0.29	-0.25
19		0.98		-1.73	1.07	-1.86		0.16		-1.22	0.29	0.20		0.52		-0.38	-0.17	-0.01		0.22		-0.63	-0.20	0.18
20	0.72	-0.79	-0.50	0.36	-0.79	-1.74	-0.20	-0.13	-0.46	-0.50	0.44	-0.17	-0.74	-0.21	-0.60	-0.45	0.49	-0.74	-0.22	-0.03	-0.42	-0.28	0.35	-0.18
	**2-hydroxyisobutyrate**						**3-aminoisobutyrate**						**3-hydroxybutyrate/3-aminoisobutyrate**						**3-hydroxyisobutyrate**					
1	-0.02		0.16	0.23	-0.21	0.30	-0.11		0.90	0.42	0.70	0.21	0.16		-0.20	0.22	-0.01	0.20	-0.05		-0.08	-0.43	0.06	0.60
2	-0.13	0.20		0.34	0.54	0.11	0.19	-0.83		-0.16	0.87	0.50	0.38	-1.00		0.54	0.32	0.04	0.09	0.16		-0.06	0.57	-0.02
3	-0.14	-0.37	-0.31	0.14	-0.33	0.13	-0.58	-1.07	-0.61	2.00	-0.07	-1.50	0.15	0.28	0.15	0.24	-0.09	-0.46	-0.18	-0.11	0.02	0.19	-0.10	-0.35
4	0.24	0.09	0.13	0.57		-0.37	0.17	-0.18	-0.06	-0.03		-0.01	0.60	-0.55	0.25	-0.02		-0.65	0.00	0.22	0.72	-0.29		0.33
5	0.35	-0.27	0.01	-0.13	-0.29	-0.02	1.23	2.00	-0.68	-0.44	-1.05	0.33	-0.11	-0.39	-0.36	-0.79	-0.08	-0.53	0.09	0.08	0.03	-0.69	-0.65	-0.35
6		0.04	-0.13	-0.17	0.15	-0.11		0.83	-0.35	-1.27	0.13	0.17		0.37	-0.35	0.06	-0.01	0.48		0.19	-0.15	0.09	0.02	0.20
7	-0.23		-0.24	-0.05	-0.24	-0.17	0.15		-0.65	1.18	0.60	-0.39	-0.44		-0.22	0.13	-0.04	0.02	-0.36		-0.17	-0.15	-0.60	0.19
8	-0.74	-0.10	0.42	0.23	0.00	-0.30	-2.00	-1.96	-0.65	0.02	0.72	-1.72	-0.27	-0.36	0.16	0.14	-0.16	-0.60	0.05	-0.43	0.30	-0.23	0.34	-0.63
9	0.15	-0.18	0.18	0.13	0.22	-0.34	-2.00	1.17	-1.10	N	-0.62	0.64	-0.78	0.49	-0.17	-0.18	0.02	-0.01	-0.21	0.58	-0.67	0.10	-0.25	0.07

10		0.36	0.23	0.10	-0.04	0.20		0.94	-0.15	-0.29	-0.25	0.27		0.28	-0.31	0.81	-0.32	-0.01		0.38	-0.71	0.46	0.38	-0.04
11	-0.33	0.49	-0.07	0.13	0.15	0.09	-0.13	-0.38	-0.93	-0.13	-0.20	-0.22	0.06	0.09	-0.20	-0.34	-0.24	0.00	0.40	-0.10	-0.04	-0.47	-0.27	0.29
12	0.01	-0.46	0.43	0.29	-0.19	0.23	0.78	-1.18	-1.66	1.87	-0.23	0.67	0.23	-0.75	0.17	-0.49	-0.09	-0.10	0.65	-0.40	-0.22	0.00	0.13	0.26
13	0.32	-0.21	-0.02	-0.21	-0.40	0.18	-0.17	-0.06	-0.42	-0.01	-1.10	0.14	-0.25	0.00	-0.12	0.18	-0.27	-0.05	-0.30	0.09	0.11	-0.07	0.02	-0.33
14	-0.14	0.01		-0.28	0.06	-0.09	-0.51	-0.71		-0.55	-0.63	1.04	-0.28	-0.25		0.25	-0.39	-0.34	0.48	-0.45		-0.25	-0.61	-0.83
15	-0.24	0.22	-0.38	0.58	-0.13	0.20	0.45	-2.00	-0.68	2.00	-1.55	1.07	0.07	-0.54	0.00	-0.72	0.51	0.13	-0.12	-0.36	-0.03	-0.12	0.36	-0.74
16	-0.36	0.11			0.00	0.10	2.00	2.00			0.44	-0.79	-0.69	-0.40			-0.10	-0.19	-0.10	-0.41			0.12	-0.44
17	0.52	0.29	0.46		-0.35	0.35	2.00	-0.04	-2.00		-1.41	1.23	0.05	0.16	0.36		0.03	0.10	0.08	-0.15	-0.19		-0.41	-0.09
18	-0.12	0.07	-0.22	-0.01	0.07	-0.37	2.00	1.15	-0.14	0.46	2.00	-0.59	0.42	-0.41	-0.03	-0.76	0.12	-0.28	-0.07	-0.71	-0.19	-0.70	-0.99	-0.38
19		-0.13		0.05	-0.01	-0.09		-1.01		-0.26	2.00	0.87		0.38		-0.71	-0.04	0.25		-0.43		-1.24	-0.21	0.17
20	0.37	0.40	0.30	-0.23	0.80	-0.21	0.34	0.29	0.74	0.47	0.07	1.01	0.24	-0.30	-0.21	-0.89	-0.19	-0.30	-0.28	-0.34	-0.87	-0.65	0.07	-0.14

	**3-hydroxyisovalerate**						**3-indoxylsulfate**						**4-deoxyerythronic acid**						**4-deoxythreonic acid**					
1	0.01		0.02	-0.20	0.59	0.23	0.92		-0.24	1.04	-0.43	0.50	0.15		0.10	0.06	0.42	0.31	0.20		0.35	0.56	0.38	0.29
2	0.26	-0.10		0.43	0.45	0.44	-0.23	-0.21		0.50	0.57	0.03	0.04	-0.17		0.42	0.78	0.33	-0.26	0.38		0.73	0.54	-0.36
3	-0.35	0.00	0.00	0.30	0.05	0.09	-0.39	1.18	-1.32	0.45	-0.10	0.09	-0.04	0.53	-0.17	0.17	0.49	-0.31	0.12	-0.01	0.55	0.09	0.13	0.25
4	0.34	0.45	0.73	-0.16		0.24	-0.71	-0.07	-0.09	0.31		0.20	1.05	0.26	1.04	-0.19		-0.77	0.35	0.65	0.59	0.55		-0.55
5	0.17	-0.21	0.00	-0.32	-0.25	-0.17	0.18	-1.07	0.16	2.00	-1.99	-0.18	0.33	-0.08	-0.12	-0.01	0.08	0.16	0.47	0.88	-0.45	-0.83	0.60	-0.10
6		0.29	-0.32	0.00	0.09	0.15		0.00	1.32	-0.61	2.00	-0.21		0.37	0.44	0.19	0.17	0.33		0.39	0.07	0.14	0.56	0.19
7	0.29		-0.31	-0.06	-0.48	0.35	0.01		-1.82	0.25	-0.43	-0.78	-0.27		-0.08	0.48	0.30	-0.04	-0.70		0.18	0.21	-0.32	0.01
8	0.13	-0.21	0.46	-0.11	0.53	-0.11	0.65	0.02	0.81	1.73	1.72	-1.37	0.44	-0.39	0.40	1.87	-0.36	-0.50	-0.97	0.10	-0.52	0.68	-0.20	0.01
9	-0.02	0.40	-0.55	0.16	0.27	0.18	-2.00	-0.51	0.28	-0.76	1.49	-2.00	0.12	0.56	-0.09	0.47	0.34	0.18	0.10	-0.28	0.00	-0.13	0.78	0.32
10		0.49	-0.21	0.28	0.51	0.23		0.53	0.01	0.73	2.00	0.59		0.62	-0.29	-0.13	0.27	0.08		0.22	-0.03	0.70	0.05	0.31
11	0.10	-0.28	-0.18	-0.54	-0.28	0.22	-0.52	1.70	-0.61	-0.19	0.14	-0.57	0.56	-0.11	0.08	-0.68	0.26	0.06	0.33	-0.10	0.13	0.22	0.18	0.40
12	0.42	-0.48	-0.16	-0.23	0.20	-0.04	0.09	-0.93	0.66	0.23	0.10	-0.64	0.42	-0.03	0.38	-0.19	0.49	-0.11	0.41	-0.83	0.69	0.08	0.48	0.23
13	-0.15	0.10	0.42	0.19	-0.08	-0.37	-0.16	-0.58	0.12	0.05	-0.40	0.91	0.08	0.44	0.41	0.10	0.09	-0.23	0.59	-0.06	0.41	0.07	-0.55	0.42
14	0.33	-0.50		0.06	-0.68	-0.61	-0.40	-0.95		1.95	-0.69	-2.00	-0.01	0.06		-0.01	-0.37	-0.46	0.63	-0.16		0.20	0.59	0.40
15	-0.26	0.51	-0.20	-0.19	0.10	-0.22	-0.45	0.13	-0.03	0.87	-0.17	-0.51	0.40	0.63	-0.17	0.13	0.67	-0.38	0.04	0.16	-0.32	0.43	0.75	0.33
16	-0.27	-0.18			0.01	-0.29	0.52	0.59			-0.28	-0.49	0.15	-0.43			-0.14	0.13	0.59	-0.43			-0.11	0.13
17	0.14	0.02	0.11		-0.24	-0.01	0.28	0.62	0.08		0.52	0.58	0.11	0.03	0.53		-0.27	-0.13	-0.48	0.54	1.41		0.20	0.92

18	-0.12	-0.39	-0.35	-0.52	-0.40	-0.28	0.02	-0.04	-0.57	0.49	-0.91	1.49	0.39	-0.05	-0.16	-0.25	0.01	0.10	-0.01	0.18	0.21	0.31	1.03	-0.01
19		0.27		-0.53	0.02	0.20		2.00		0.89	0.19	-0.48		0.51		-0.59	0.41	0.57		0.49		0.24	0.44	0.37
20	-0.17	-0.08	-0.52	-0.19	0.29	-0.38	-0.18	-0.15	0.60	1.41	0.56	-1.05	-0.13	0.16	-0.19	-0.23	0.05	0.49	0.32	0.68	0.66	0.33	0.88	0.45
